# A Coupled Eulerian–Lagrangian Framework for Tracing Dynamic Response of Polymer-Based Composite Laminates Under Close-In and Near-Field Spherical Explosions

**DOI:** 10.3390/polym18212630

**Published:** 2026-10-23

**Authors:** Debjyoti Roychowdhury, Dipti Ranjan Sahoo, Shamsher Bahadur Singh, Sudhirkumar V. Barai, Venkatesh Kodur

**Affiliations:** 1 Department of Civil Engineering, IIT Delhi, Hauz Khas, New Delhi 110016, India; debjyoti.roychowdhury@civil.iitd.ac.in (D.R.); drsahoo@civil.iitd.ac.in (D.R.S.); 2 Department of Civil Engineering, Dean Infrastructure and Planning, BITS Pilani, Pilani Campus, Pilani 333031, India; sbsingh@pilani.bits-pilani.ac.in; 3 Department of Civil Engineering, Pilani Campus, Pilani 333031, India; skbarai@pilani.bits-pilani.ac.in; 4 Civil and Environmental Engineering (CEE), Michigan State University, 426 Auditorium Road, East Lansing, MI 48824, USA

**Keywords:** blast, coupled Eulerian–Lagrangian, finite element analysis, laminate, first-order shear deformation

## Abstract

Polymer composites are lightweight materials with superior stiffness and elastic characteristics, making them well suited for aeronautical structures under complex loading. This study investigates the dynamic response of FRP composite plates subjected to close-in and near-field spherical blasts using a Coupled Eulerian–Lagrangian (CEL) framework. Quarter-symmetric finite element models are developed to reduce computational cost while maintaining computational accuracy. Fluid–structure interaction is simulated using appropriate state equations for air and explosive media. Orthotropic, strain-rate-dependent material properties from standard literature are adopted, and a representative volume element approach is adopted to define the material direction in each ply to form laminate. The entire numerical framework for blast conditions has been validated in modules against benchmark studies, showing good agreement. Various laminated stiffener configurations are designed to evaluate the effect of bottom stiffening on blast resistance. Parametric studies examine the influence of layer numbers, stiffener thickness, and stiffener depth under close-in blast conditions. Results indicate that, for the specific cross- and angle-ply laminate configurations as investigated, for a constant laminate thickness, an increase in ply count is associated with a reduction in peak deflection. Further, within the scope of stiffener geometries as studied, variation in stiffener thickness resulted in a more pronounced reduction in blast-loaded FRP plate response than the corresponding variation in stiffener depth.

## 1. Introduction

The design of blast-resistant protective structures has become increasingly important due to the growing frequency of blast incidents and their severe consequences for national security, human safety, and critical infrastructure. In aeronautical and defence applications, the development of lightweight blast-resistant structures has attracted considerable attention because of the microstructural complexities involved in achieving high strength-to-weight performance. Furthermore, the design of their critical components requires explicit consideration of plastic deformation to ensure reliable structural performance under blast loading.

Composite materials are among the most versatile engineered materials for advanced structural applications owing to their low density, high stiffness, and superior specific mechanical properties. These attributes provide excellent strength-to-weight performance, making composites particularly suitable for lightweight blast-resistant structures. Their efficient use in protective enclosure walls subjected to internal blast loading can significantly reduce structural weight while maintaining impact resistance, thereby offering considerable potential for aeronautical applications, including aircraft baggage compartments and cargo containers.

Blast-induced structural response and damage have traditionally been investigated using isotropic homogeneous and heterogeneous materials, including metals and concrete. Structural strengthening has also relied on conventional materials, particularly steel; however, these materials offer limited potential for lightweight design. The emergence of fibre-reinforced polymer (FRP) composites, characterized by their superior specific mechanical properties, has shifted research towards lightweight blast-resistant structures. Consequently, extensive numerical, analytical, and experimental studies have investigated the blast response of FRP laminates, both as primary structural components and as retrofit materials for the strengthening of existing structures. Nam et al. (2008) [1] evaluated three material formulations for FRP laminates: isotropic linear elastic shell (IES); orthotropic linear elastic shell (OES) with and without transverse shear deformation; and linear elastic beam (EB) models using finite element-based framework. Following validation of the blast analysis methodology, these formulations were assessed by comparing the blast response of FRP sheets and FRP-retrofitted two-way reinforced concrete slabs using LS-DYNA v.970 (LSTC, 2003). A comprehensive understanding of FRP behaviour under blast loading, however, requires detailed investigation of blast-induced damage and the associated energy dissipation mechanisms. Accordingly, several studies have focused on quantifying and characterizing blast-induced damage in FRP laminate structures. Batra and Hassan (2007) [2] developed an in-house finite element code to investigate blast-induced damage in four-layered FRP laminates by modelling fibre–matrix debonding, fibre breakage, and matrix cracking in the time domain. Delamination between adjacent layers was evaluated based on stress states satisfying the composite failure criterion while allowing multiple damage mechanisms to occur simultaneously within the laminate. The study also quantified the energy absorbed through each failure mode. In addition to their use as primary structural components, FRP composites have been widely employed as retrofit patches to enhance the blast resistance of existing structures. Pereira et al. (2010) [3] investigated the use of CFRP patches to repair blast-damaged steel box sections through combined experimental and numerical analyses of three cracked configurations. The CFRP patches effectively reduced the stress concentration around the cracks, thereby suppressing crack propagation and extending the service life of the steel structures.

Beyond steel structures, FRP laminate retrofitting has also been widely employed to enhance the blast resistance of reinforced concrete and composite wall systems. Anas and Alam (2022) [4] investigated the use of 0.15 mm thick carbon-fibre-reinforced polymer (CFRP) laminates to strengthen concrete walls against blast-induced bulging under 10, 15, and 20 kg TNT charges at a close stand-off distance of 0.5 m. In addition to single-layer CFRP, the study evaluated multilayer CFRP configurations for high-intensity blast scenarios using the ABAQUS/Explicit-v.6.15 solver. Besides CFRP, aramid fibre-reinforced polymer (AFRP or Kevlar FRP) laminates have also been employed to retrofit reinforced concrete columns, thereby enhancing the blast resistance of these critical load-bearing members and reducing the likelihood of progressive structural collapse.

Researchers have also worked on strengthening such columns using FRP retrofits under blast scenarios. Hosseini et al. (2025) [5] numerically investigated the blast performance of reinforced concrete (RC) columns retrofitted with CFRP and AFRP using the LS-DYNA finite element code. The influence of FRP layer configuration on the blast resistance of the columns was also examined. Both retrofit systems effectively mitigated blast-induced damage and reduced residual displacement; however, AFRP exhibited superior performance over CFRP in terms of mid-span displacement and energy absorption. Similar investigations have also been conducted on CFRP-retrofitted clay brick masonry walls subjected to blast loading. Shariq and Alam (2022) [6] numerically investigated the blast resistance of clay brick masonry walls retrofitted with CFRP laminates under a 5 kg TNT free-air explosion at a scaled distance of 0.58 m/kg^1/3^. The retrofitting configurations included 0.5 and 0.6 mm thick CFRP laminates applied to the rear face and a 0.3 mm thick laminate applied to both faces of the wall. The study also evaluated steel wire mesh reinforcement with diameters of 2.5, 3.5, and 4.5 mm. The results showed that a 4.5 mm diameter wire mesh applied to both faces and a 0.5 mm thick CFRP laminate applied to the rear face provided comparable blast resistance.

To simulate blast loading representative of field conditions, researchers have employed both analytical formulations and numerical methods. Analytical approaches generate blast pressure histories using empirical decay functions, whereas numerical simulations typically adopt Lagrangian, Eulerian, or Arbitrary Lagrangian–Eulerian (ALE) multi-material formulations. However, the applicability of these methods depends on the blast characteristics and scaled distance. These approaches have been widely used to investigate the response and damage of FRP laminates and FRP-retrofitted structures subjected to blast loading. Tran et al. (2016) [7] numerically investigated the deformation and failure of multilayer elastomer/fibre-reinforced polymer (FRP) composite panels subjected to blast loading. The blast load was idealized as a uniformly distributed triangular pressure pulse with a peak overpressure of 49.3 MPa and a positive phase duration of 25 μs. E-glass/vinylester laminates were modelled using the Hashin damage criterion to capture fibre and matrix failure as well as interlaminar delamination. A thin polyurea backing layer was further incorporated to mitigate blast-induced damage. Hosseini et al. (2023) [8] numerically investigated the blast performance of full-scale RC slabs strengthened with hybrid FRP laminates using the LS-DYNA finite element framework. Blast loading was modelled using the Load Blast Enhanced (CONWEP) option based on the Kingery–Bulmash empirical blast model with TNT equivalence following James et al. (2008) [9]. Carbon and glass fibres were combined in the hybrid laminate, and a laminated composite material model was adopted to predict composite damage. The influence of laminate stacking sequence on the damage response of FRP-retrofitted RC slabs was also examined, demonstrating that hybridization of the outermost ply reduced the damaged area. Similar Lagrangian-based blast modelling techniques have also been employed to evaluate the blast resistance of FRP-retrofitted reinforced concrete columns. Zhang and Abedini (2021) [10] numerically investigated the blast response of FRP-retrofitted reinforced concrete (RC) columns using the LS-DYNA finite element framework. Blast loading was applied through the Load Blast Enhanced (CONWEP) formulation, which empirically generates pressure–time histories from the explosive charge weight and stand-off distance and was validated against the experimental results of Abedini et al. (2020) [11]. The study highlighted the limitation of the CONWEP approach in neglecting air–structure interaction, restricting its applicability to close-in blast scenarios. Pressure–impulse (P–I) diagrams and a damage index were further developed to quantify blast-induced damage and residual load-carrying capacity while assessing the influence of FRP wrap thickness, strength, and configuration.

Similar numerical approaches have also been employed to investigate the blast response of concrete-filled FRP tube structures using constitutive models for explosive materials. Qasrawi et al. (2015) [12] numerically investigated the close-in blast response of concrete-filled fibre-reinforced polymer tubes (CFFTs) using the ANSYS Autodyn 2005 framework. The study employed the Jones–Wilkins–Lee (JWL) equation of state for TNT and C4 explosives, while blast-wave propagation through air was modelled using the ideal gas equation of state. A parametric study examining tube diameter, reinforcement ratio, and blast severity was conducted to develop pressure–impulse (P–I) diagrams for the tested CFFT specimens. Similar studies have also evaluated empirical blast pressure formulations through validation against experimental observations. Mutalib and Hao (2010) [13] numerically investigated the blast performance of FRP-strengthened reinforced concrete (RC) walls with and without end anchorage systems, considering bond behaviour, anchorage performance, and premature debonding. The study showed that end anchorages effectively prevented FRP peeling and enhanced blast resistance, although localized stress concentrations could initiate FRP rupture. To account for the non-uniform spatial distribution of blast pressure, the wall was discretized into segments and segment-specific pressures were determined using the empirical formulations of Wu and Hao (2004) [14], consistent with TM5-1300 blast loading specifications. The predicted pressure histories were subsequently validated against the field tests (Muszynski and Purcell 2003 [15]).

Experimental studies have investigated the response of FRP-wrapped RC columns subjected to near-ground blast loading, with particular emphasis on reflected blast pressures. Complementary numerical models were developed to reproduce the experimental observations and extended through parametric investigations. Li et al. (2021) [16] developed a finite element model to investigate the blast response of FRP-retrofitted reinforced concrete (RC) columns and validated the predicted structural response against the field blast tests conducted by Karagozian & Case (K&C) (Crawford et al. 2012, 2013; Morrill et al. 2012) [17,18,19]. Recognizing the absence of measured pressure–time histories and the complexity of capturing ground-reflected blast waves through empirical formulations, the authors employed the AUTODYN hydrocode to simulate blast loading, explicitly accounting for ground reflection. The generated pressure histories were subsequently applied to the structural model to evaluate blast-induced damage while incorporating strain-rate-dependent behaviour of concrete, reinforcing steel, and FRP. Yen et al. (2019) [20] employed an Arbitrary Lagrangian–Eulerian (ALE) finite element framework to simulate near-field shock-hole blast loading. The blast model incorporated the Jones–Wilkins–Lee (JWL) equation of state for the C4 explosive and a linear polynomial equation of state for air. The numerical framework was validated against ten near-field shock-hole blast experiments conducted by the Transportation Security Laboratory (Nansteel and Chen 2014 [21]) on twelve-ply CFRP panels, demonstrating the suitability of the adopted explosive and air material models for close-range blast simulations.

Although extensive research has investigated the blast response and damage of FRP laminates and FRP-retrofitted structures, studies on the blast performance of FRP laminates stiffened with laminated composite or sandwich stiffeners remain limited. Türkmen and Mecitoğlu (1999) [22] experimentally and numerically investigated the response of stiffened laminated composite plates subjected to normal blast loading generated in a cylindrical shock tube using an LPG–O_2_ mixture. Finite element predictions of strain histories and peak strains were validated against experimental measurements, with the observed discrepancies attributed to the adhesive layer between the plate and the stiffener. Balkan et al. (2020) [23] developed an analytical model to investigate the nonlinear dynamic response of laminated composite plates stiffened with composite sandwich stiffeners under non-uniform blast loading. The blast pressure history, obtained and validated using shock-tube experiments, was incorporated into a meshless weighted residual formulation based on the Galerkin method. Geometric nonlinearity was accounted for using the von Kármán large-deformation theory, and the governing coupled equations were solved to predict the blast-induced displacements, strains, and stresses.

Researchers have also explored diverse applications of composite materials under blast loading and developed computational frameworks for high-impact, high-velocity problems. These studies span marine structures, blast-fragment analysis, and predictive modelling of explosive materials using equation-of-state-based detonation models. Xie et al. (2009) [24] investigated the application of composite structures in marine engineering owing to their high specific stiffness, strength, and impact resistance. The authors proposed a coupled Eulerian–Lagrangian (CEL) framework for simulating the two-dimensional and axisymmetric response of deformable composite structures subjected to blast and shock loading by coupling a Eulerian multiphase fluid solver with a Lagrangian structural solver. The proposed approach was demonstrated through the analysis of an underwater explosion acting on a three-layer composite structure. Price et al. (2015) [25] proposed a point-particle modelling framework, coupled with a multi-material Eulerian solver, to predict the acceleration and terminal velocity of explosion-driven spherical fragments. The approach was applied to fragments at different locations relative to the explosive surface, and the influence of explosive confinement was further examined using a cylindrical C4 charge. Wang et al. (2015) [26] developed a computational fluid–structure interaction framework for highly nonlinear structural response and dynamic fracture under compressible flow loading. The method enforced fluid–structure transmission conditions by solving local one-dimensional Riemann problems at embedded structural interfaces. Its applicability was demonstrated for blast- and shock-related problems involving detonation, large pressure and density discontinuities, structural fragmentation, and flow through fracture paths. Esen et al. (2005) [27] developed a predictive framework for characterizing the non-ideal detonation behaviour of commercial explosives using the JWL++ hydrocode and the DeNE detonation model. The JWL++ employed a two-dimensional Arbitrary Lagrangian–Eulerian formulation, whereas DeNE was based on a pseudo-one-dimensional theory. Following validation against measured in-hole detonation velocities, the models were used to predict key detonation parameters, including velocity, pressure, specific volume, reaction extent, and reaction-zone length. Rabb and Fahrenthold (2010) [28] developed a hybrid particle–element formulation to simulate projectile impact and perforation in woven fabrics. The model was validated against experimental results for aramid and ultra-high-molecular-weight polyethylene (UHMWPE) soft armour, demonstrating good agreement in predicting impact-induced fragmentation.

Research on the blast response of FRP composites and FRP-strengthened structures has expanded owing to their high specific strength and energy absorption capability. However, compared with conventional homogeneous and heterogeneous structures, the blast response and post-blast damage of FRP laminates remain relatively underexplored. In particular, the effects of laminated composite and sandwich stiffeners on blast resistance, together with validated fluid–structure interaction frameworks incorporating appropriate equation-of-state models for TNT and air, have received limited attention. Moreover, the response of FRP laminates under close-in (<0.4 m/kg^1/3^) and near-field free-air blast loading has received limited attention. Addressing these gaps is important for the development of effective design strategies for lightweight blast-resistant containment structures in aeronautical applications such as aircraft baggage compartments, where internal explosions generally produce close-in or near-field blast loading. As a preliminary step towards such systems, this study develops and validates a Coupled Eulerian–Lagrangian (CEL) framework against published experimental data and applies it to investigate the dynamic response of laminated FRP composite plates representing enclosure side walls. The validated model is subsequently used to evaluate the blast response of unstiffened and bottom-stiffened laminates and to assess the effectiveness of different stiffener configurations. A comprehensive parametric study is performed to quantify the effects of ply number at constant laminate thickness, stacking sequence, and bottom-mounted stiffener configuration on the transient peak deflection and blast resistance of FRP laminates under close-in and near-field blast loading. Accordingly, the objectives of the present study are to: (a) develop a finite element-based coupled Eulerian–Lagrangian (CEL) framework for simulating close-in and near-field blast loading and capturing blast pressure histories, (b) establish a fluid–structure interaction model incorporating appropriate equation-of-state descriptions for explosive and air, (c) evaluate the effectiveness of different laminated composite bottom-stiffener configurations, and (d) identify the governing parameters for enhancing the blast resistance of stiffened FRP laminates.

## 2. Stress–Strain Constitutive Relation and Force Field

In this section, the governing mathematical formulations are described as part of micro- and macro-mechanics of the laminates. The governing equations are formulated within a systematic framework to define the stresses in individual laminae through stress–strain constitutive relations based on micromechanics of laminates and are subsequently used to compute the force resultants in a laminate.

**Figure 1:**
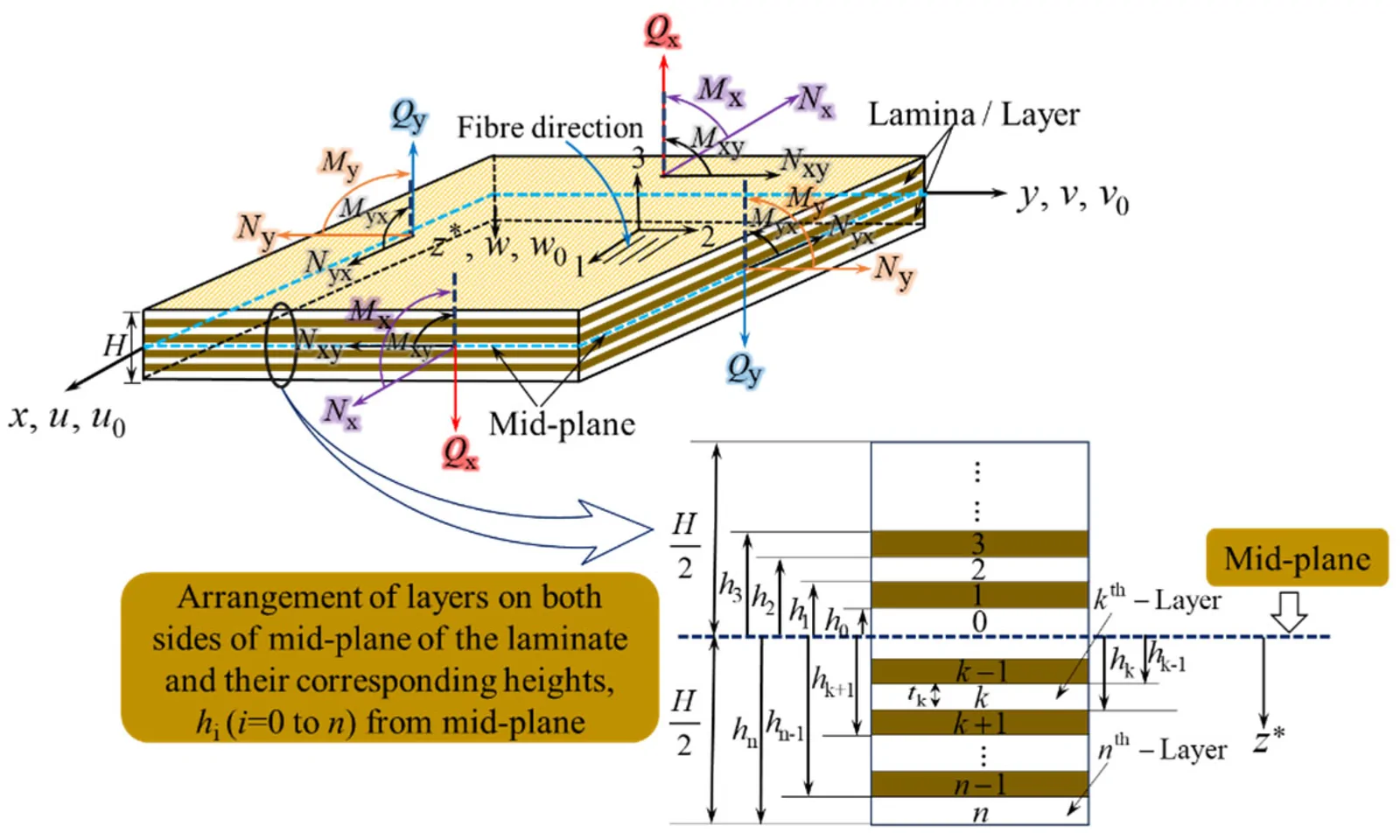
Schematic diagram of FRP laminated composite plate and the associated force vectors.

This section presents the governing formulations based on the micro- and macro-mechanics of laminated composites. The constitutive relations are first established to evaluate the lamina stresses from the stress–strain relationships and are subsequently integrated through the laminate thickness to obtain the laminate force resultants. Based on its aspect ratio and maximum deflection criterion (Shwetha et al. 2018 [29]), the laminate considered in this study is classified as thick. Accordingly, the material model is formulated within the framework of the first-order shear deformation theory (FSDT), also known as the Mindlin–Reissner theory (Reissner 1945 [30]), to account for transverse shear deformation. Figure 1 illustrates the laminate geometry, material coordinate system, and lamina positions with respect to the mid-plane. The mid-surface displacements comprise the mid-plane displacement components $u_{0}\left(x,\;y\right)$ in the x direction and $v_{0}\left(x,\;y\right)$ in the y direction, constant transverse displacement $w_{0}$ along thickness $z^{*}$. Under the FSDT assumption, the in-plane axial displacements $u\left(x,\;y,z^{*}\right)$ and $v\left(x,\;y,z^{*}\right)$ vary linearly across laminae through thickness $z^{*}$, while the transverse displacement $w\left(x,\;y\right)$ remains constant along $z^{*}$.

Equation (1) defines the in-plane normal and shear strains in the $k^{\mathrm{th}}$ orthotropic lamina of a laminated composite. According to the first-order shear deformation theory (FSDT), the in-plane strains are related to the laminate mid-plane strains ($\varepsilon_{x}^{0},\;\varepsilon_{y}^{0},\;\gamma_{\mathrm{xy}}^{0},\;\gamma_{{\mathrm{yz}}^{*}}^{0},\;\gamma_{{\mathrm{xz}}^{*}}^{0}$) and curvature components ($\kappa_{x},\;\kappa_{y},\;\kappa_{\mathrm{xy}}$) involving mid-plane axial rotations $\psi_{x}\left(x,\;y\right)$ and $\psi_{y}\left(x,\;y\right)$ resulting in a linear variation in strains ($\varepsilon_{x},\;\varepsilon_{y},\;\gamma_{\mathrm{xy}},\;\gamma_{{\mathrm{yz}}^{*}},\;\gamma_{{\mathrm{xz}}^{*}}$) with the lamina position $z^{*}$ through thickness H (Sreehari 2017 [31]). The strain field is expressed in the following matrix form:

(1) $${\left[\begin{array}{c} \varepsilon_{x}\\ \varepsilon_{y}\\ \gamma_{\mathrm{xy}}\\ \begin{array}{l} \gamma_{{\mathrm{yz}}^{*}}\\ \gamma_{{\mathrm{xz}}^{*}} \end{array} \end{array}\right]}_{k}={\left[\begin{array}{c} \frac{\partial u_{0}}{\partial x}\\ \frac{\partial v_{0}}{\partial y}\\ \frac{\partial u_{0}}{\partial y}+\frac{\partial v_{0}}{\partial x}\\ \begin{array}{l} \frac{\partial w_{0}}{\partial y}+\psi_{y}\\ \frac{\partial w_{0}}{\partial x}+\psi_{x} \end{array} \end{array}\right]}_{k}+z^{*}{\left[\begin{array}{c} \frac{\partial \psi_{x}}{\partial x}\\ \frac{\partial \psi_{y}}{\partial y}\\ \frac{\partial \psi_{x}}{\partial y}+\frac{\partial \psi_{y}}{\partial x}\\ \begin{array}{l} 0\\ 0 \end{array} \end{array}\right]}_{k}$$

The right-hand side of Equation (1) consists of a mid-plane strain vector:

(2) $$\left\{\begin{array}{c} \varepsilon_{x}^{0}\\ \varepsilon_{y}^{0}\\ \gamma_{\mathrm{xy}}^{0}\\ \begin{array}{l} \gamma_{{\mathrm{yz}}^{*}}^{0}\\ \gamma_{{\mathrm{xz}}^{*}}^{0} \end{array} \end{array}\right\}=\left\{\begin{array}{c} \frac{\partial u_{0}}{\partial x}\\ \frac{\partial v_{0}}{\partial y}\\ \frac{\partial u_{0}}{\partial y}+\frac{\partial v_{0}}{\partial x}\\ \begin{array}{l} \frac{\partial w_{0}}{\partial y}+\psi_{y}\\ \frac{\partial w_{0}}{\partial x}+\psi_{x} \end{array} \end{array}\right\}$$

and the mid-plane curvatures:

(3) $$\left\{\begin{array}{l} \kappa_{x}\\ \kappa_{y}\\ \kappa_{\mathrm{xy}} \end{array}\right\}=\left\{\begin{array}{c} \frac{\partial \psi_{x}}{\partial x}\\ \frac{\partial \psi_{y}}{\partial y}\\ \frac{\partial \psi_{x}}{\partial y}+\frac{\partial \psi_{y}}{\partial x} \end{array}\right\}$$

Since each lamina is orthotropic, its material axes generally differ from the laminate global coordinate system. Accordingly, the local constitutive relations are transformed into the global coordinates. Incorporating transverse shear deformation, the constitutive relation for the $k^{\mathrm{th}}$ lamina (Thai and Choi 2013 [32]) is expressed as

(4) $${\left\{\begin{array}{c} \sigma_{x}\\ \sigma_{y}\\ \tau_{\mathrm{xy}}\\ \tau_{{\mathrm{yz}}^{*}}\\ \tau_{{\mathrm{xz}}^{*}} \end{array}\right\}}_{k}={\left[\begin{array}{ccccc} {\bar{Q}}_{11} & {\bar{Q}}_{12} & {\bar{Q}}_{16} & 0 & 0\\ {\bar{Q}}_{11} & {\bar{Q}}_{22} & {\bar{Q}}_{26} & 0 & 0\\ {\bar{Q}}_{16} & {\bar{Q}}_{26} & {\bar{Q}}_{66} & 0 & 0\\ 0 & 0 & 0 & {\bar{Q}}_{44} & {\bar{Q}}_{45}\\ 0 & 0 & 0 & {\bar{Q}}_{45} & {\bar{Q}}_{55} \end{array}\right]}_{k}{\left\{\begin{array}{c} \varepsilon_{x}\\ \varepsilon_{y}\\ \gamma_{\mathrm{xy}}\\ \gamma_{{\mathrm{yz}}^{*}}\\ \gamma_{{\mathrm{xz}}^{*}} \end{array}\right\}}_{k}.$$

In Equation (4), ${\bar{Q}}_{\mathrm{ij}}$ denotes the transformed reduced stiffness coefficients relating the stress and strain components of the $k^{\mathrm{th}}$ lamina. Integrating these stiffness coefficients through the thickness of each lamina ($t_{k}$) and summing their contributions over the laminate yields the laminate stiffness matrices in Equation (5), which define the in-plane axial force ($N_{x},\;N_{y},\;N_{\mathrm{xy}}$), bending moment ($M_{x},\;M_{y},\;M_{\mathrm{xy}}$) and transverse shear resultants ($Q_{x},\;Q_{y}$). The transverse shear stiffness components are evaluated using the shear correction factor (α), which compensates for the difference between the actual stress state and the assumed constant stress state (Thai and Choi 2013 [32]). Accordingly, the laminate force and moment resultants are related to the mid-plane strains and curvatures through the following matrix equation:

(5) $$\left\{\begin{array}{c} N_{x}\\ N_{y}\\ N_{\mathrm{xy}}\\ M_{x}\\ M_{y}\\ M_{\mathrm{xy}}\\ Q_{x}\\ Q_{y} \end{array}\right\}=\left[\begin{array}{cccccccc} A_{11} & A_{12} & A_{16} & B_{11} & B_{12} & B_{16} & 0 & 0\\ A_{12} & A_{22} & A_{26} & B_{12} & B_{22} & B_{26} & 0 & 0\\ A_{16} & A_{26} & A_{66} & B_{16} & B_{26} & B_{66} & 0 & 0\\ B_{11} & B_{12} & B_{16} & D_{11} & D_{12} & D_{16} & 0 & 0\\ B_{12} & B_{22} & B_{26} & D_{12} & D_{22} & D_{26} & 0 & 0\\ B_{16} & B_{26} & B_{66} & D_{16} & D_{26} & D_{66} & 0 & 0\\ 0 & 0 & 0 & 0 & 0 & 0 & \bar{A_{44}} & \bar{A_{45}}\\ 0 & 0 & 0 & 0 & 0 & 0 & \bar{A_{45}} & \bar{A_{55}} \end{array}\right]\left\{\begin{array}{c} \varepsilon_{x}^{0}\\ \varepsilon_{y}^{0}\\ \gamma_{\mathrm{xy}}^{0}\\ \kappa_{x}\\ \kappa_{y}\\ \kappa_{\mathrm{xy}}\\ \gamma_{{\mathrm{yz}}^{*}}^{0}\\ \gamma_{{\mathrm{xz}}^{*}}^{0} \end{array}\right\}$$

where $A_{\mathrm{ij}}$, $B_{\mathrm{ij}}$, and $D_{\mathrm{ij}}$ are the extensional, extensional-bending, and bending stiffness matrix terms of matrices $\left[A\right]$, $\left[B\right]$, and $\left[D\right]$, respectively, determined using Equations (6)–(8):

(6) $$A_{\mathrm{ij}}={\sum_{k=1}^{n}\int_{h_{k-1}}^{h_{k}}\left[\bar{Q_{\mathrm{ij}}}\right]}_{k}dz^{*},\mathrm{where}\;\int_{h_{k-1}}^{h_{k}}dz^{*}=(h_{k}-h_{k-1}),\;i=1,\;2,\;6;\;j=1,\;2,\;6,$$

(7) $$B_{\mathrm{ij}}=\frac{1}{2}{\sum_{k=1}^{n}\int_{h_{k-1}}^{h_{k}}\left[\bar{Q_{\mathrm{ij}}}\right]}_{k}z^{*}dz^{*},\mathrm{where}\;\int_{h_{k-1}}^{h_{k}}dz^{*}=\frac{1}{2}(h_{k}^{2}-h_{k-1}^{2}),\;i=1,\;2,\;6;\;j=1,\;2,\;6,$$

(8) $$D_{\mathrm{ij}}=\frac{1}{3}{\sum_{k=1}^{n}\int_{h_{k-1}}^{h_{k}}\left[\bar{Q_{\mathrm{ij}}}\right]}_{k}z^{*2}dz,\mathrm{where}\;\int_{h_{k-1}}^{h_{k}}dz^{*}=\frac{1}{3}(h_{k}^{3}-h_{k-1}^{3}),\;i=1,\;2,\;6;\;j=1,\;2,\;6,$$

(9) $$\bar{A_{\mathrm{pq}}}={\sum_{k=1}^{n}\int_{h_{k-1}}^{h_{k}}\alpha \left[\bar{Q_{\mathrm{pq}}}\right]}_{k}dz^{*},\mathrm{where}\;\int_{h_{k-1}}^{h_{k}}dz^{*}=(h_{k}-h_{k-1}),\;p=4,\;5;\;q=4,\;5.$$

Equation (9) defines the transverse shear stiffness matrix $\left[\bar{A}\right]$, incorporating the shear correction factor (α). In Equation (5), $\left[A\right]$ relates in-plane forces to the mid-plane strains, $\left[B\right]$ couples the force and moment resultants to the mid-plane strains and mid-plane curvatures, $\left[D\right]$ relates the bending and torsional moment resultants to laminate’s mid-plane curvatures, and $\left[\bar{A}\right]$ forms the relation between the resultant transverse shear forces and mid-plane transverse shear strains (Kaw 2005 [33]; Thai and Choi 2013 [32]).

## 3. Finite Element Modelling of Composite Laminate Under Blast Load

### 3.1. Model Details

**Figure 2:**
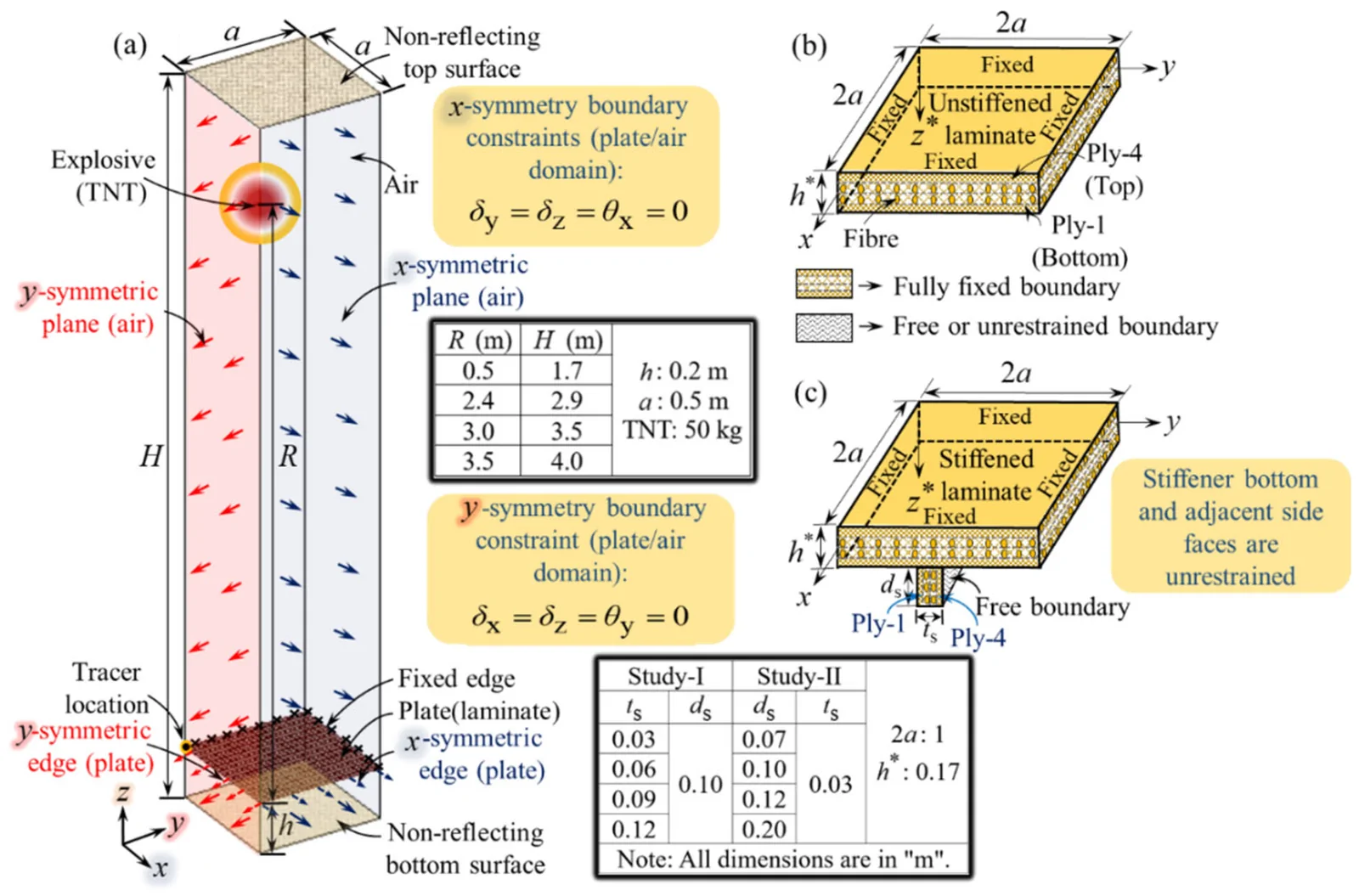
(**a**) Quarter-symmetric CEL model and schematics of (**b**) unstiffened and (**c**) stiffened laminates.

This section presents the finite element modelling of laminated composite plates subjected to close-in and near-field blast loading. Four laminate configurations—symmetric cross-ply ${\left[0/90\right]}_{s}$, symmetric angle-ply ${\left[\pm 45\right]}_{s}$, unsymmetric cross-ply ${\left[0/90\right]}_{2}$, and unsymmetric angle-ply ${\left[\pm 45\right]}_{2}$—with dimensions of 1 × 1 × 0.17 m were analysed using LS-DYNA R12 finite element code by implementing coupled Eulerian–Lagrangian (CEL) framework with fully clamped edges. Blast loading was generated by a 50 kg TNT charge at stand-off distances of 0.5, 2.4, 3.0, and 3.5 m, corresponding to scaled distances of 0.14, 0.65, 0.81, and 0.95 m/kg^1/3^, representing one close-in (0.14 < 0.4 m/kg^1/3^, Orton et al. 2013 [34]; Raftenberg 1997 [35]) and three near-field blast events (0.65, 0.81, 0.95 < 1.18 m/kg^1/3^, Paccou et al. 2026 [36]). To improve computational efficiency, quarter-symmetric models were adopted with appropriate symmetry boundary conditions applied to both the Eulerian air domain and the Lagrangian laminate. The spherical explosive charge is introduced as a Eulerian volume fraction using the INITIAL_DETONATION card. Laminates were modelled using four-node shell elements with a mesh size of 20 mm, using MAT_COMPOSITE_LAYUP for material property and PART_COMPOSITE_LONG to define the layup sequence. The air model is discretized using eight-noded Eulerian solid (air) elements of size 0.02 × 0.02 × 0.005 m. The air depth (H) is optimized to 1.7, 2.9, 3.5, and 4.0 m for the respective stand-off distances through mesh convergence studies, while the laminate position (h) within the air domain was maintained at 0.2 m from the bottom boundary. Figure 2 illustrates the quarter-symmetric CEL model adopted for analysing both unstiffened and stiffened laminates. Blast performance is evaluated using four bottom-mounted FRP laminated stiffener configurations, modelled with four-noded thin shell elements (Louca et al. 1998 [37]; Galiev 1996 [38]) with FE discretization of 25 × 10 while employing the same laminate lay-up procedure as in the parent plate. The corresponding boundary conditions for each stiffener configuration are summarized in Table 1.

Previous studies have predominantly investigated stiffened composite plates with stiffeners mounted on the blast-facing surface, directly exposed to the incident shock wave. Interestingly, the profile of a shock wave changes with time (Paccou et al. 2026 [36]). In contrast, the present study proposes bottom-mounted laminated composite stiffeners to examine their effectiveness in enhancing blast resistance. Four stiffener configurations are considered: S1 (unidirectional), S2 (bidirectional), S3 (diamond-frame), and S4 (diagonal), as illustrated in Table 1. The laminate lay-up is defined sequentially from the bottom ply (ply-1 as per Figure 2) following the loading direction. The connection between stiffener(s) with a laminated plate has been modelled implementing a tie mechanism. Such a connection algorithm initially ties the connected surfaces or edges together, facilitating monolithic load transfer between the stiffener(s) and laminated plate. When the prescribed interface failure criterion is reached, the tied connection can progressively lose its load-transfer capability, which can result in interface damage/debonding. Following the failure of the tied interface, the connection formulation permits in building surface-to-surface contact to prevent physical penetration between the stiffener(s)’ and laminate’s connecting the surface(s) or edge(s). Alternatively, a popular modelling approach applicable for shell elements is monolithic modelling of common nodes along the connecting edge(s) of the stiffener(s) and plate.

As shown in Figure 2a, a quarter-symmetric coupled Eulerian–Lagrangian (CEL) model is adopted to reduce computational cost while maintaining solution accuracy. The Lagrangian laminate and Eulerian air domain were modelled separately and coupled through a fluid–structure interaction algorithm. Appropriate symmetry boundary conditions were applied to preserve the physical response of the full-scale model.

**Figure 3:**
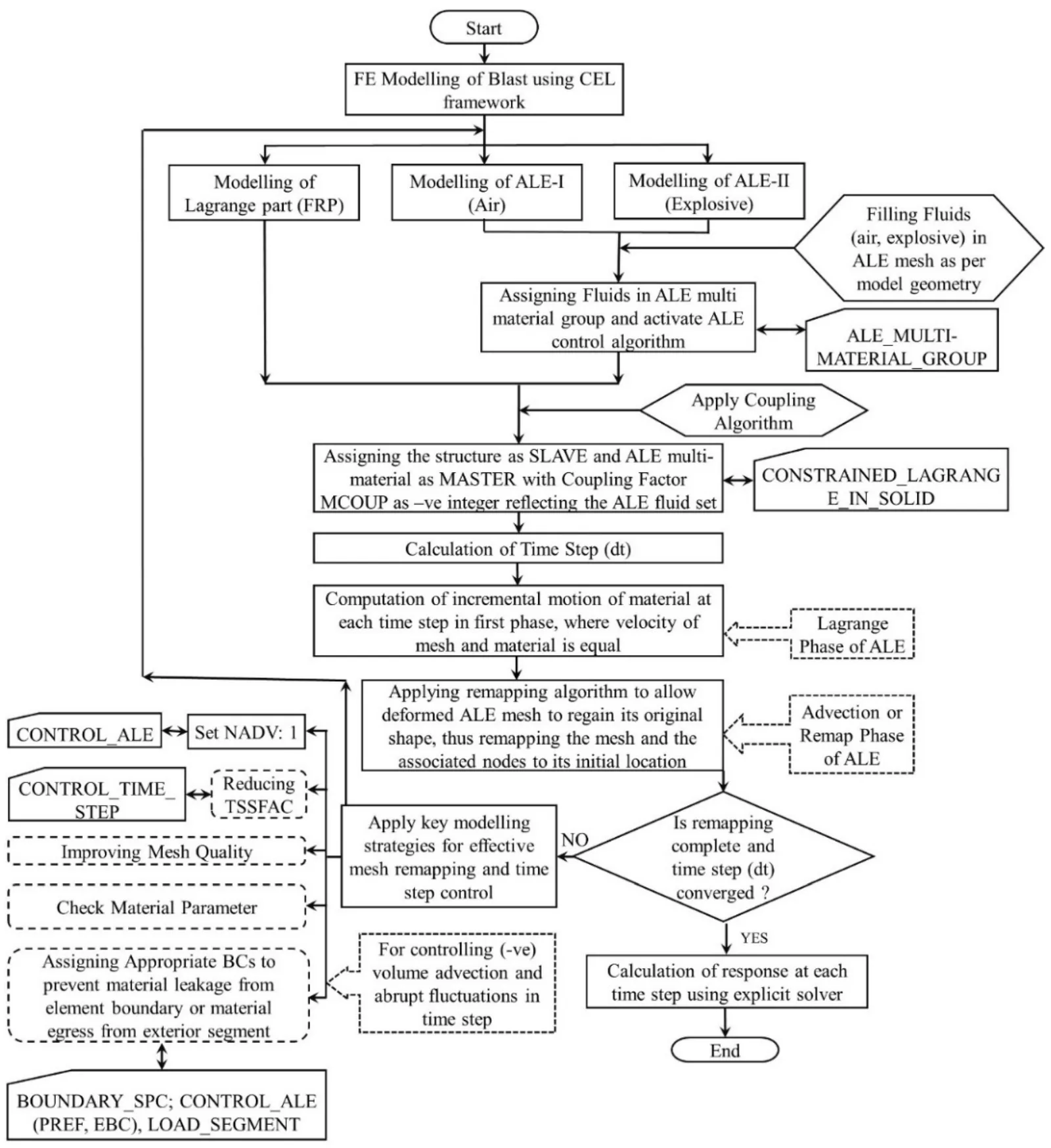
Process flow diagram of the CEL model adopted in simulating blast scenarios. (a) One-end arrow: Indicates flow; (b) Both-end arrow: Indicates alternative options; (c) Dotted arrow end: Indicates occurrence of “Lagrange” and “Advection” phases.

Figure 3 outlines the coupled Eulerian–Lagrangian (CEL) framework adopted to simulate the blast events. The explosive and air were modelled as an ALE multi-material domain, whereas the laminate was represented using Lagrangian elements. Appropriate numerical controls were implemented to mitigate negative-volume advection and time-step convergence issues commonly encountered in blast simulations, thereby ensuring stable execution of the coupled fluid–structure interaction algorithm. To maintain coupling stability and prevent material leakage across the Eulerian–Lagrangian interface, two coupling points were assigned along each ALE element edge. Owing to the adopted numerical formulation, vent openings were not considered in the computational domain.

For close-in (Schwer et al. 2015 [39], Shi et al. 2022 [40]) blast simulations, leakage-control parameters (PFAC, FRCMIN, NORMTYPE, and ILEAK) were carefully optimized to prevent explosive material leakage across the Eulerian–Lagrangian interface. Fluid–structure interaction was modelled using the penalty-coupling algorithm (Knight and Qi 1997 [41]; Orton et al. 2013 [34]), with the laminate defined as the slave component and the Eulerian fluids (air and explosive) as the master component. All simulations were performed using a consistent LS-DYNA unit system (kg, m, s, and Pa).

### 3.2. Material Model

#### 3.2.1. Explosive Model

The explosive was modelled in LS-DYNA using the MAT_HIGH_EXPLOSIVE_BURN material model (Hallquist 2003 [42]; Hallquist 1992 [43]), with the spherical TNT charge defined through the INITIAL_VOLUME_FRACTION_GEOMETRY option. The detonation products were described using the Jones–Wilkins–Lee (JWL) equation of state (Lee et al. 1973 [44]; Dobratz 1981 [45]; Giam et al. 2020 [46]), which relates the blast pressure to the relative explosive volume and internal energy, thereby governing the release and propagation of the blast wave. The JWL equation is expressed as

(10) $$p=A\left(1-\frac{\omega }{R_{1}V}\right)e^{-R_{1}V}+B\left(1-\frac{\omega }{R_{2}V}\right)e^{-R_{2}V}+\frac{\omega E}{V},$$

where ω, $R_{1}$, $R_{2}$, A, and B are user-defined input parameters. The explosive material property and the supporting state parameters used to simulate the explosion are summarized in Table 2.

#### 3.2.2. Air Model

The air domain was modelled using the MAT_NULL material model (Hallquist 2003 [42]; Hallquist 1992 [43]), which is commonly employed with high-explosive formulations by neglecting material strength (Lee et al. 1973 [44]; Dobratz 1981 [45]; Giam et al. 2020 [46]). Accordingly, the viscous stress ($\sigma^{\prime }$) in air is assumed to be proportional to the deviatoric strain rate (${\dot{\varepsilon }}^{\prime }$) (Shin et al. 2014 [47]; Liu et al. 2021 [48]; Yuan et al. 2023 [49]) and is expressed as

(11) $$\sigma^{\prime }=\mu {\dot{\varepsilon }}^{\prime },$$

where μ is the dynamic viscosity coefficient (Pa-s). The MAT_NULL model is coupled with the EOS_LINEAR_POLYNOMIAL equation of state (Hallquist 2003 [42]; Hallquist 1992 [43]), whose parameters satisfy the gamma-law equation to accurately represent the thermodynamic behaviour of air during blast-wave propagation. The corresponding pressure ($p^{*}$) is expressed as

(12) $$p^{*}=C_{0}+C_{1}\mu^{\prime }+C_{2}{\mu^{\prime }}^{2}+C_{3}{\mu^{\prime }}^{3}+\left(C_{4}+C_{5}\mu^{\prime }+C_{6}{\mu^{\prime }}^{2}\right)E,$$

where $C_{i}$ (i = 0 to 6) are the equation constants and $\mu^{\prime }=\frac{\rho_{0}}{\rho }-1$; $\frac{\rho }{\rho_{0}}$ or $\frac{V}{V_{0}}$ is relative density or relative volume. ρ, V are actual density and volume at any time (t); $\rho_{0},\;V_{0}$ refer the nominal density and volume at t=0 s. Equation (12) defines ambient air pressure in polynomial form. E is the internal energy/volume, and an initial value of ${\left.E\right|}_{t=0}$ or $E_{0}$ is used as a state parameter input to simulate the behaviour of air. The properties of air and its applied state parameters are consolidated in Table 3.

#### 3.2.3. Composite Laminate Model

The composite laminates were modelled using the MAT_54/MAT_116 material model (Hallquist 2003 [42]; Hallquist 1992 [43]), which defines the fibre, matrix, and ply orientations. The adopted material properties are summarized in Table 4, which describes the orthotropic elastic properties of the laminate, including the longitudinal and transverse Young’s moduli ($E_{1}$ and $E_{2}$), in-plane shear modulus ($G_{12}$), transverse shear moduli (*G*_23_ and *G*_13_), Poisson’s ratios ($\nu_{12}$ and $\nu_{21}$), and density (ρ). The minor Poisson ratio ($\nu_{21}$), is determined from the major Poisson ratio ($\nu_{12}$) through the reciprocal relation of $\nu_{21}=\nu_{12}\frac{E_{1}}{E_{2}}$. The ρ is evaluated using the rule of mixtures. Fibre orientation is specified through the local material coordinate system defined in the material card.

The accuracy of the proposed finite element framework was established through comprehensive validation studies. Close-in blast pressure and structural response were validated against published experimental data (Appendix A.1 and Appendix A.3), whereas near-field blast pressure histories were compared with the UFC 3-340-02 benchmark (Sreehari 2017 [31]; Shin et al. 2014 [47]) predictions (Appendix A.2). The responses of unstiffened and stiffened FRP laminates under blast and impulsive loading were further validated against published analytical solutions (Appendix A.4 and Appendix A.5).

## 4. Results from Numerical Study

### 4.1. Blast Pressure and Impulse Study

Blast pressure was recorded using the DATABASE_TRACER option with the THIST algorithm enabled through the ASCII database. The tracer, acting as a virtual pressure sensor, was fixed in the computational domain to capture the incident blast pressure while minimizing the influence of reflected waves. An unstiffened-${\left[0/90\right]}_{s}$ laminate model, configured according to Figure 2b, was analysed under different stand-off distances, with the tracer located at the plate centre to record the peak pressure–time histories.

**Figure 4:**
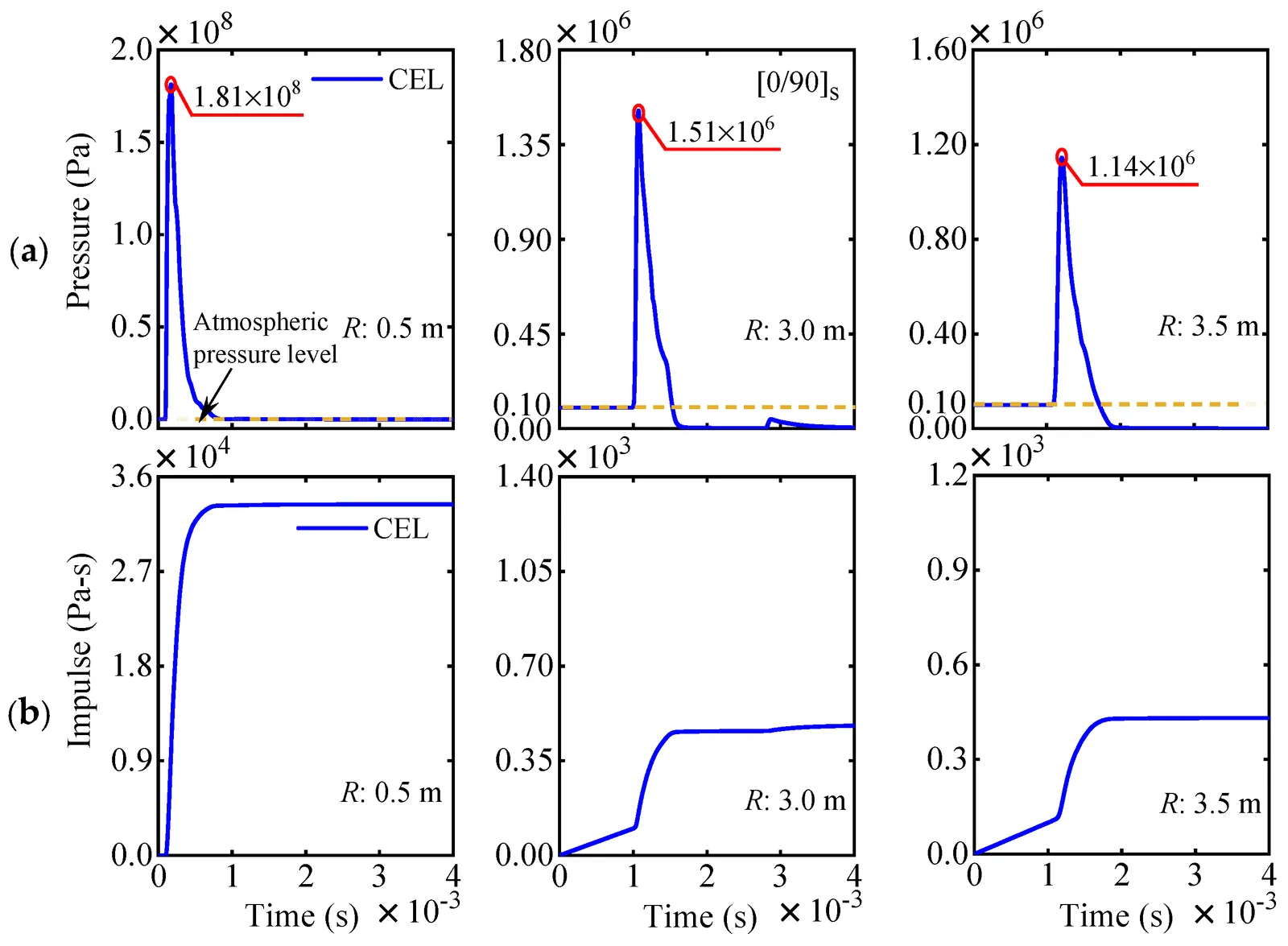
(**a**) Blast pressure relative to ambient atmospheric pressure level and (**b**) impulse time history data obtained under different blast scenarios.

Figure 4 presents the predicted blast pressure- and impulse-time histories at the plate centre for different blast scenarios. Under close-in loading, the peak pressure is attained at 0.17 ms after detonation, whereas for near-field blasts at R 3.0 and 3.5 m, the corresponding peak pressures occur at 1.07 and 1.21 ms, respectively. The close-in blast generates a peak pressure of 1.81 × 10^8^ Pa, approximately 99% higher than those for 3.0 and 3.5 m stand-off cases. The corresponding peak impulse reaches 3.34 × 10^4^ Pa-s under close-in blast, while the peak impulses of 4.79 × 10^2^ Pa-s and 4.32 × 10^2^ Pa-s are observed for R at 3.0 and 3.5 m, respectively. With a constant charge weight of 50 kg TNT, both peak pressure and impulse decrease with increasing scaled distance.

### 4.2. Blast-Induced Stresses at the Top and Bottom Surfaces of the Composite Laminates

**Figure 5:**
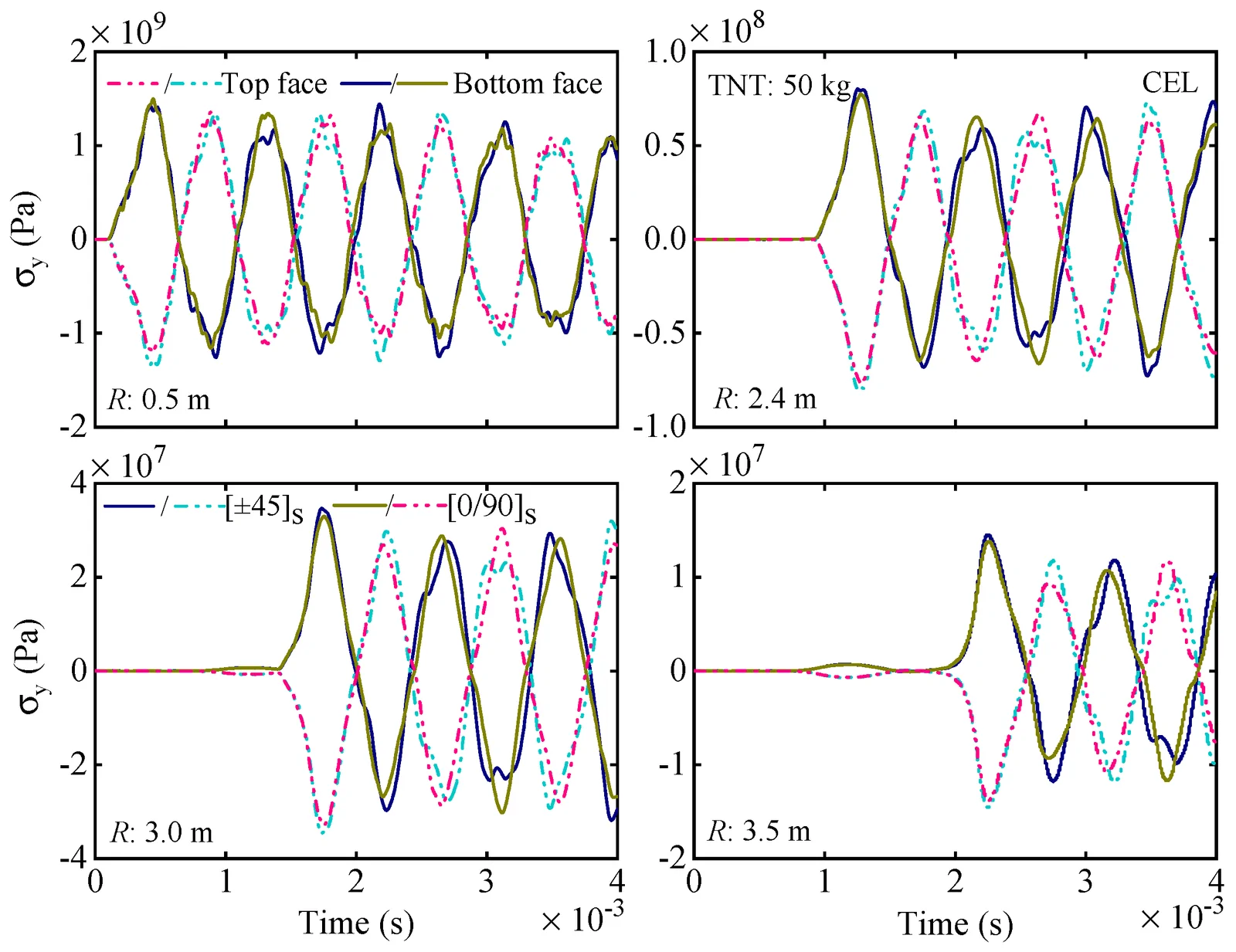
Blast-induced normal bending stress at the top and bottom face of cross and angle ply laminates.

The blast-induced bending stresses at the top and bottom surfaces of unstiffened four-layered ${\left[0/90\right]}_{s}$ and ${\left[\pm 45\right]}_{s}$ are evaluated for four blast scenarios of R = 0.5, 2.4, 3.0, and 3.5 m using the plate configuration shown in Figure 2b. Since the laminate response is periodic, the two surfaces experience alternating tensile and compressive stresses. The peak normal bending stress in the global y direction, $\sigma_{y}$, is captured at the plate centre and presented in Figure 5, while the corresponding peak values are summarized in Table 5. For both laminates, the difference between the peak stresses at the top and bottom surfaces decreases with increasing R, becoming nearly equal and opposite at R = 3.5 m, indicating an approximately linear through-thickness stress distribution under near-field blast loading. The ${\left[0/90\right]}_{s}$ exhibits lower peak bending stresses than the ${\left[\pm 45\right]}_{s}$ for near-field cases like R = 2.4, 3.0, and 3.5 m. Under the close-in blast (R: 0.5 m), as compared to ${\left[\pm 45\right]}_{s}$, ${\left[0/90\right]}_{s}$ develops a lower peak stress at the top surface but a marginally (0.06 GPa) higher bending stress experienced on the bottom surface. Overall, the cross-ply configuration demonstrates superior blast resistance, owing to the absence of extensional–shear and bending–twisting coupling, resulting in reduced deflections and bending stresses. Furthermore, the peak stresses remain well below the corresponding elastic moduli (Table 4), indicating elastic behaviour under all considered blast scenarios.

### 4.3. Unstiffened Laminated Composite Plates Subjected to Close-In and Near-Field Blast

**Figure 6:**
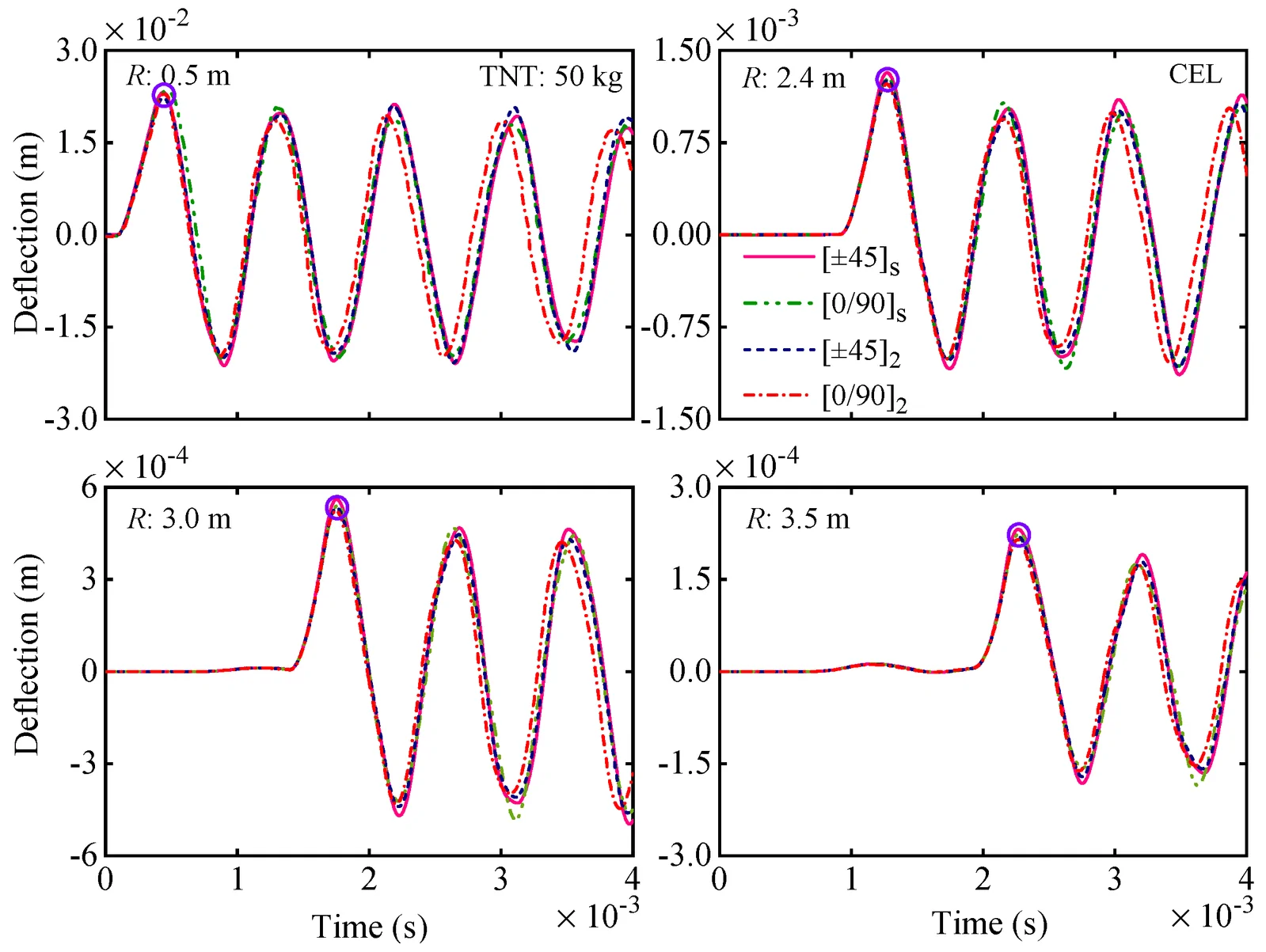
Deflection profiles of clamped unstiffened laminates under close-in and near-field blasts. Occurrence of blast-induced peak deflections for laminates have been marked with circle in the deflection time-history profile.

**Figure 7:**
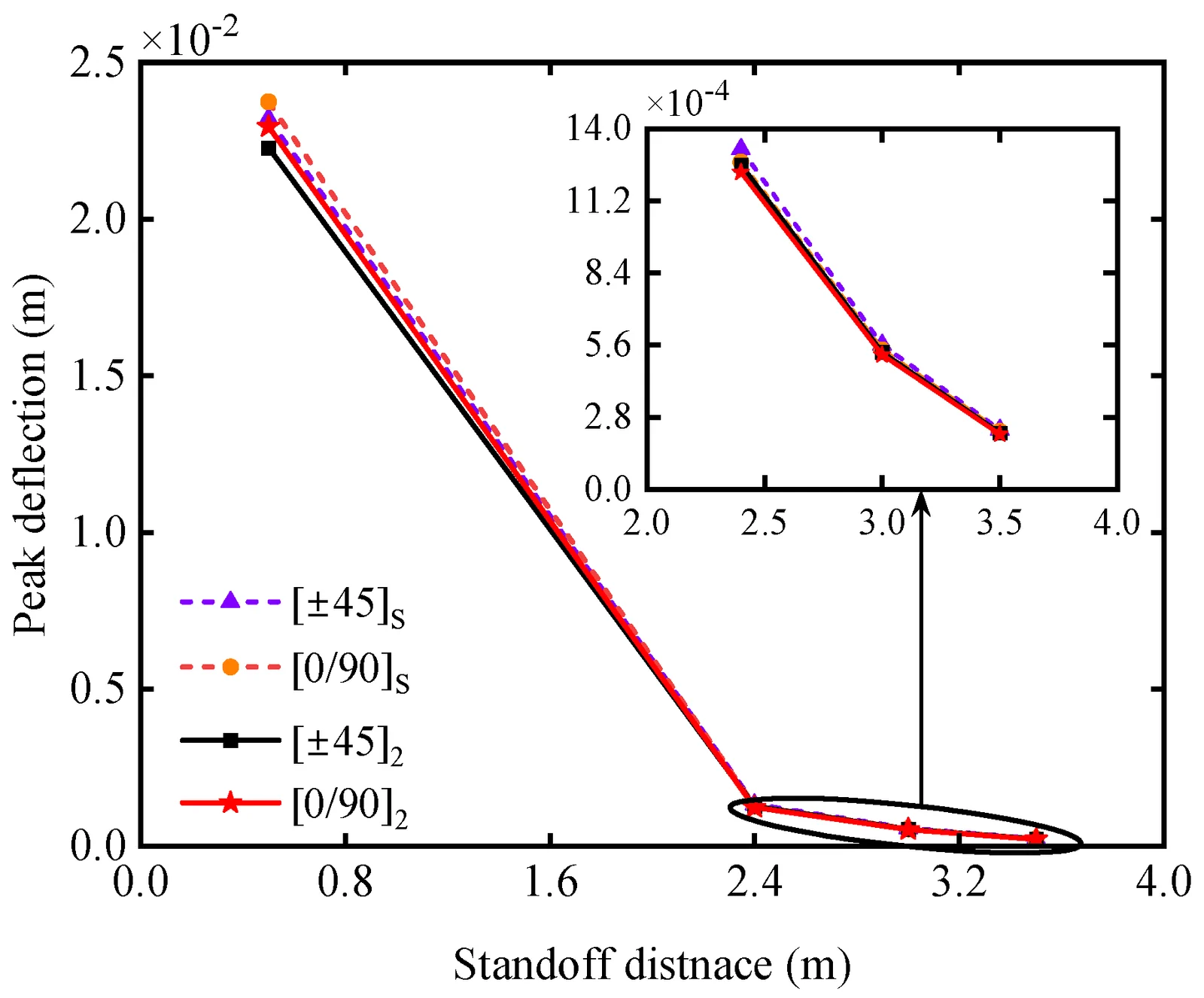
Linear variation in peak deflections of unstiffened laminates with increase in standoff distance.

This section evaluates the dynamic response of unstiffened ${\left[0/90\right]}_{s}$, ${\left[\pm 45\right]}_{s}$, ${\left[0/90\right]}_{2}$, and ${\left[\pm 45\right]}_{2}$ laminates by comparing the peak centre deflections under 50 kg TNT blast events at stand-off distances of 0.5, 2.4, 3.0, and 3.5 m. Figure 6 presents the corresponding deflection–time histories and identifies the peak deflections, while Figure 7 compares the linear variation in peak deflections with R for all laminates. Under the close-in blast, the unstiffened-${\left[\pm 45\right]}_{2}$ laminate exhibits the highest blast resistance, producing the lowest peak deflection of 0.0223 m, whereas ${\left[0/90\right]}_{2}$ laminate shows a marginally higher value of 0.0230 m. In contrast, extension–shear and bending–twisting uncoupling in the cross-ply configuration become dominant in maximizing blast resistance under near-field blast conditions (R: 3.0, 3.5 m), enabling the unstiffened-${\left[0/90\right]}_{2}$ to achieve the lowest deflection.

Further investigation is conducted to analyse the occurrence time of peak deflection for the unstiffened laminates for three blast conditions of R = 0.5, 3.0, and 3.5 m. Table 6 summarises the peak deflections and their corresponding occurrence times for the three blast events. The results clearly demonstrate that with increasing R, the temporal separation between the occurrence of peak-induced pressure and the attainment of peak deflection of the laminates widens.

### 4.4. Effect of Layer Count on the Response of Laminates Under Close-In Explosion

**Figure 8:**
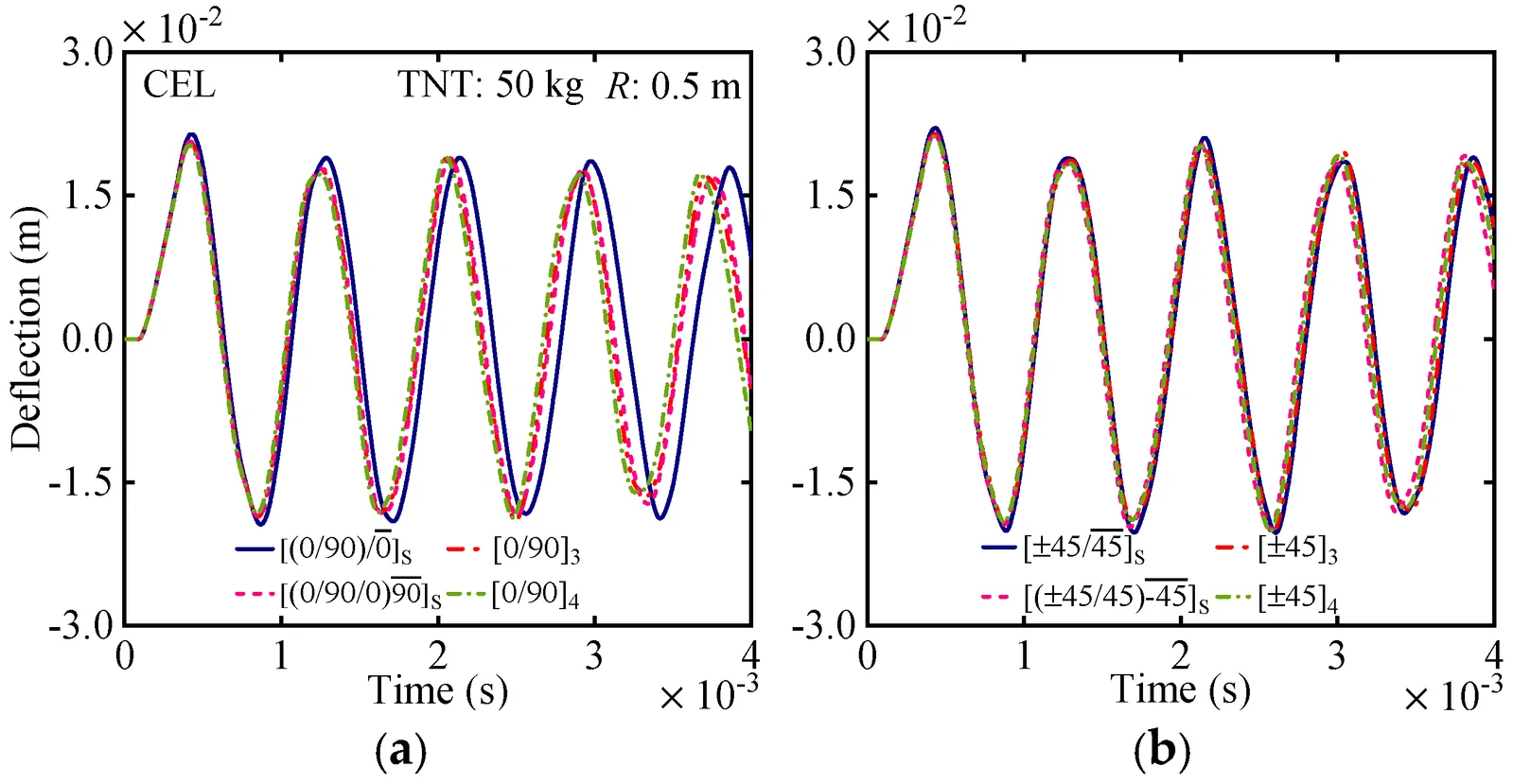
Close-in blast-induced deflection of 170 mm thick unstiffened (**a**) cross and (**b**) angle ply.

The numerical models of unstiffened-[0/90] and [±45] laminates with five, six, seven, and eight laminas, while maintaining a constant overall thickness of 170 mm, are developed to investigate the influence of layer count on the dynamic response under close-in blast. Figure 8 compares the central deflection histories, and the corresponding peak deflections are summarized in Table 7. The laminate code and corresponding stacking sequences are listed in Table 8. For both laminate configurations, increasing the number of laminas enhances blast resistance, leading to a progressive reduction in peak deflection. Relative to the four-layered ${\left[\pm 45\right]}_{s}$, the peak deflection decreases by 4.91%, 7.59%, 8.06%, and 8.75% for five, six, seven, and eight laminas, respectively. Although increasing the layer count minimizes the axial–shear ($A_{12}$, $A_{26}$) and bending–twisting ($D_{16}$, $D_{26}$) coupling terms in the stiffness matrix (Kaw 2005 [33]) of [±45] and [0/90], the [0/90] exhibits a greater reduction in peak deflections by 9.77%, 11.54%, 13.14%, and 14.74% relative to the 0.0238 m peak deflection of four-layered ${\left[0/90\right]}_{s}$. It is observed that for the present laminates, for a constant laminate thickness, the uncoupling of normal–shear forces and bending–twisting moments exhibits a pronounced effect on resultant deflections of [0/90] as compared to [±45] when the ply count increases.

From Table 7, it is quite noticeable that the uncoupling of force and moment resultants in [0/90] significantly improves its performance over [±45] under close-in detonation, demonstrating lesser deflection considering the effect of an increase in layer count for a constant laminate thickness.

### 4.5. Effect of Variations in Stiffener Geometry in Close-In Blast-Induced Response of Laminates

#### 4.5.1. Response of Stiffened Laminates Under Close-In Explosion Considering Variation in Stiffener Thickness

This section examines the dynamic response of stiffened laminates with stiffener thicknesses ($t_{s}$) of 30, 60, 90, and 120 mm while maintaining a constant stiffener depth ($d_{s}$) of 100 mm under close-in blast. For a stiffened plate geometry, boundary conditions are maintained as per Figure 2, and four stiffener configurations (Table 1) are considered for four laminate layups: ${\left[0/90\right]}_{s}$, ${\left[\pm 45\right]}_{s}$, ${\left[0/90\right]}_{2}$, and ${\left[\pm 45\right]}_{2}$. Blast resistance is evaluated by comparing the reduction in peak deflection of the stiffened laminates with the corresponding unstiffened configurations. Since the S2 configuration consistently provides the greatest reduction in peak deflection, the response comparisons are presented primarily for this stiffener design. In addition, all laminate configurations exhibit progressively lower peak deflections with increasing $t_{s}$.

${\left[0/90\right]}_{2}$ shows 11.59%, 16.91%, 21.66%, and 25.80% reductions in peak deflections against $t_{s}$ = 30 mm, 60 mm, 90 mm, and 120 mm, respectively, as compared to 0.02295 m deflection obtained the unstiffened condition. While ${\left[\pm 45\right]}_{2}$ shows 4.76%, 9.29%, 13.69%, and 18% relative to deflection (unstiffened) of 0.02228 m; ${\left[0/90\right]}_{s}$ exhibits 6.95%, 13.23%, 18.58%, and 23.21% relative to 0.02374 m deflection (unstiffened); and ${\left[\pm 45\right]}_{s}$ demonstrates 4.22%, 8.49%, 12.76%, and 17.03% reductions against the laminate’s 0.0232 m peak deflection (unstiffened) under close-in blast condition.

**Figure 9:**
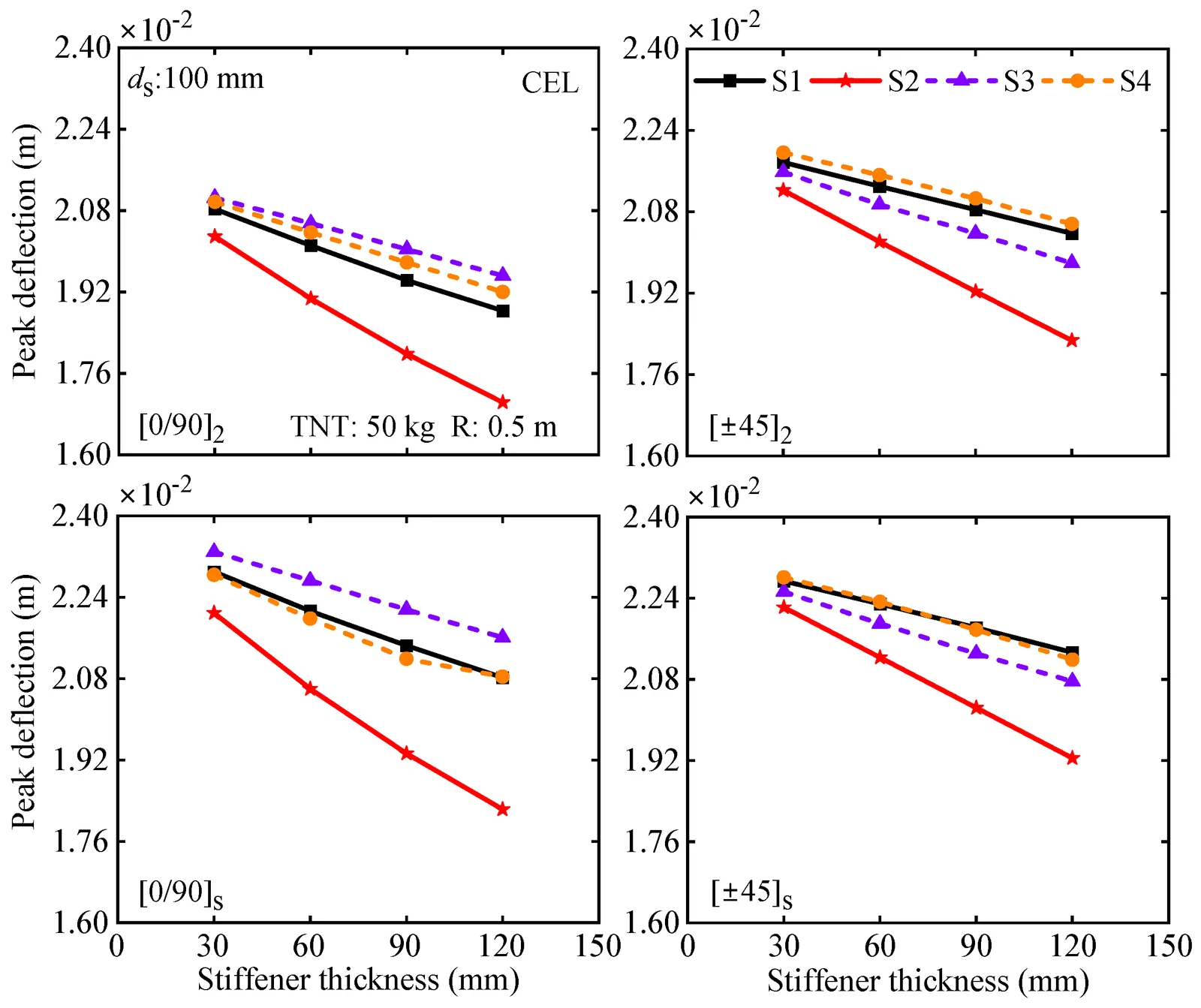
Linear variation in peak deflection of laminates with $t_{s}$ under close-in blast at $d_{s}$ 100 mm.

Figure 9 shows the linear variation in peak deflection of the stiffened laminates with $t_{s}$ for a constant $d_{s}$ of 100 mm under a close-in blast event. For all the laminate types, the S2 configuration predominantly enhances the blast resistance performance. As compared to other stiffener types, a sharp decline in peak deflection amplitudes of the stiffened laminates is experienced with the S2 configuration with an increase in $t_{s}$. However, the S3 configuration appears to be the poorest performing among all the stiffener designs in mitigating the blast load when used in combination with ${\left[0/90\right]}_{2}$ and ${\left[0/90\right]}_{s}$, while it demonstrates an average performance in the case of ${\left[\pm 45\right]}_{2}$ and ${\left[\pm 45\right]}_{s}$ considering the variation in $t_{s}$ under the close-in blast exposure condition.

#### 4.5.2. Response of Stiffened Laminates Under Close-In Explosion Considering Variation in Stiffener Depth

This section investigates the dynamic response of stiffened laminates for $d_{s}$ = 70, 100, 120, and 200 mm at a constant $t_{s}$ of 30 mm under close-in detonation. Here also, ${\left[0/90\right]}_{s}$, ${\left[\pm 45\right]}_{s}$, ${\left[0/90\right]}_{2}$, and ${\left[\pm 45\right]}_{2}$ are analysed for plate geometry and the boundary conditions shown in Figure 2 with four stiffener configurations (Table 1). Since the S2 configuration consistently provides the greatest reduction in peak deflection, the response comparisons are presented primarily for this stiffener design, which exhibits progressively improved blast resistance with increasing $d_{s}$.

${\left[0/90\right]}_{2}$ exhibits peak response reductions of 8.32%, 11.59%, 13.29%, and 21.57% for $d_{s}$ = 70 mm, 100 mm, 120 mm, and 200 mm, respectively, relative to the peak deflection of the laminate under the unstiffened condition. Similarly stiffened-${\left[\pm 45\right]}_{2}$ shows 1.23%, 4.76%, 7.14%, and 19.21%; ${\left[0/90\right]}_{s}$ exhibits 3.12%, 7%, 9.18%, and 15.75%; and 0.86%, 4.22%, 6.5%, and 13.79% reductions in peak response are observed for ${\left[\pm 45\right]}_{s}$ relative to the peak response under the unstiffened condition.

**Figure 10:**
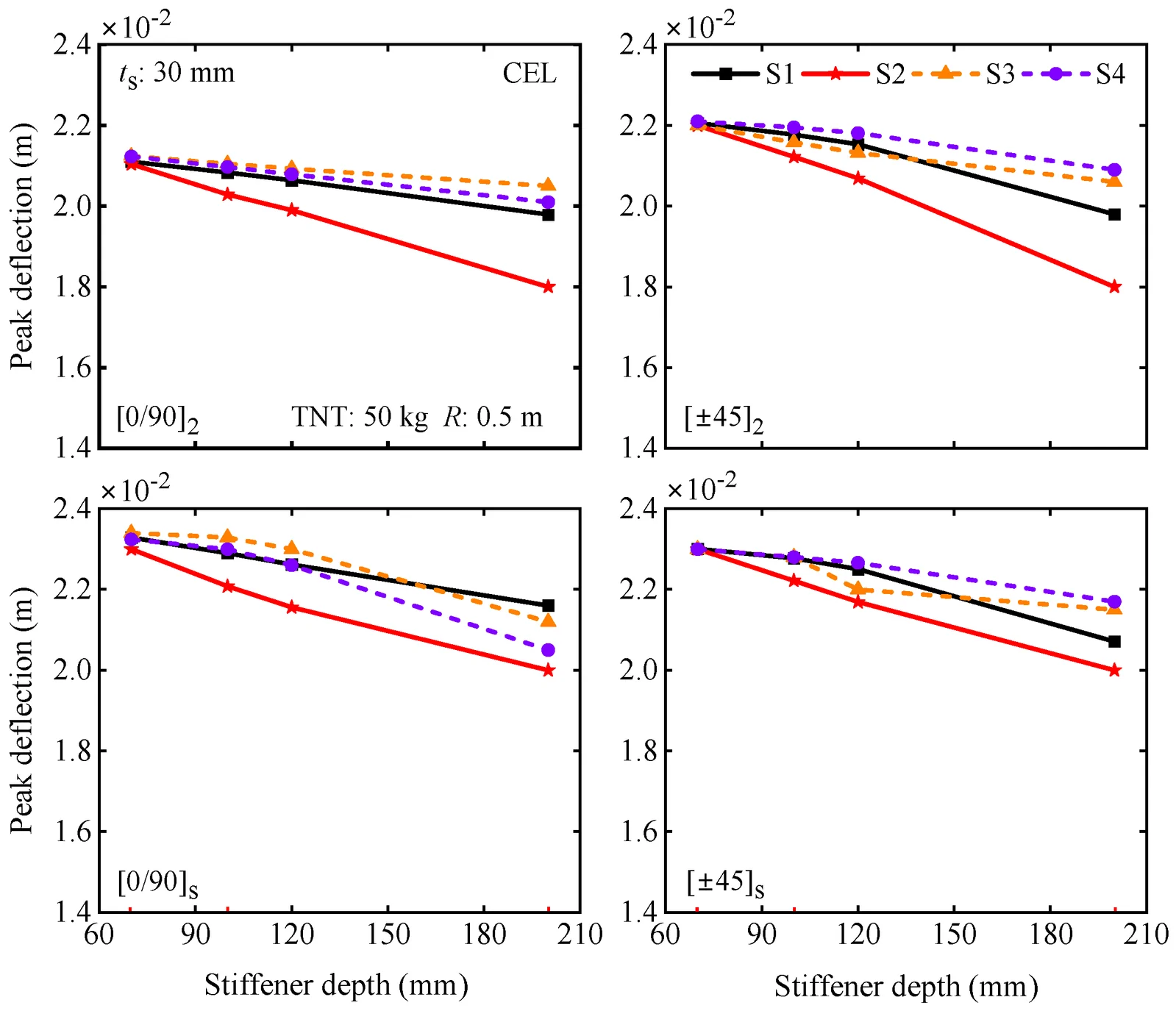
Linear variation in peak deflection of laminates with $d_{s}$ under close-in blast at $t_{s}$ = 30 mm.

Figure 10 explains the linear variation in peak deflection amplitude with $d_{s}$ for an individual laminate configuration, considering all four stiffener designs under a close-in explosion event. A sharp decline in peak deflection is observed with the S2 stiffener type for all lamina sequences, indicating the S2 configuration outperforms other stiffener designs in delivering maximum blast resistance under the close-in blast scenario. Although the S3 stiffener configuration appears to be the poorest performing in reducing peak blast-induced response for ${\left[0/90\right]}_{2}$, the same conclusion cannot be drawn for the ${\left[0/90\right]}_{s}$ laminate. The study further concludes that the S4 configuration exhibits the poorest performance in mitigating the blast load when used in combination with both the laminates (${\left[\pm 45\right]}_{2}$ and ${\left[\pm 45\right]}_{s}$) while varying $d_{s}$ under the close-in blast exposure condition.

For all laminate configurations, the S2 stiffener provides the greatest reduction in peak deflection. Since ${\left[0/90\right]}_{2}$ exhibits the largest benefit from stiffening, its response is used to quantify the impact of stiffener geometries relative to 22.95 mm peak deflection in the unstiffened case. Results indicate that with a constant $d_{s}$ of 100 mm, S2 reduces peak deflection by 11.59%, 16.91%, 21.66%, and 25.80%, respectively, while increasing $t_{s}$ from 30 to 120 mm. In contrast, for a constant $t_{s}$ of 30 mm, the same S2 yields comparatively smaller reductions of 8.32%, 11.59%, 13.29%, and 21.57%, respectively, while increasing $d_{s}$ from 70 to 200 mm. Also, the peak response reduction achieved with a maximum $t_{s}$ of 120 mm at $d_{s}$ = 100 mm is 94.10% greater than that obtained with $t_{s}$ =30 mm and $d_{s}$ = 120 mm. Similar trends are observed for the remaining laminate configurations as well. This indicates that, given the nature of the present simulated close-in blast and the lamina stacking sequences, $t_{s}$ is a more critical parameter than $d_{s}$ in influencing blast-induced peak responses of stiffened laminates as investigated under the present research scope.

### 4.6. Blast Pressure and Deflection Contours of Laminate

#### 4.6.1. Distribution of Blast Pressure over Laminate near Peak Pressure Occurrence Time

**Figure 11:**
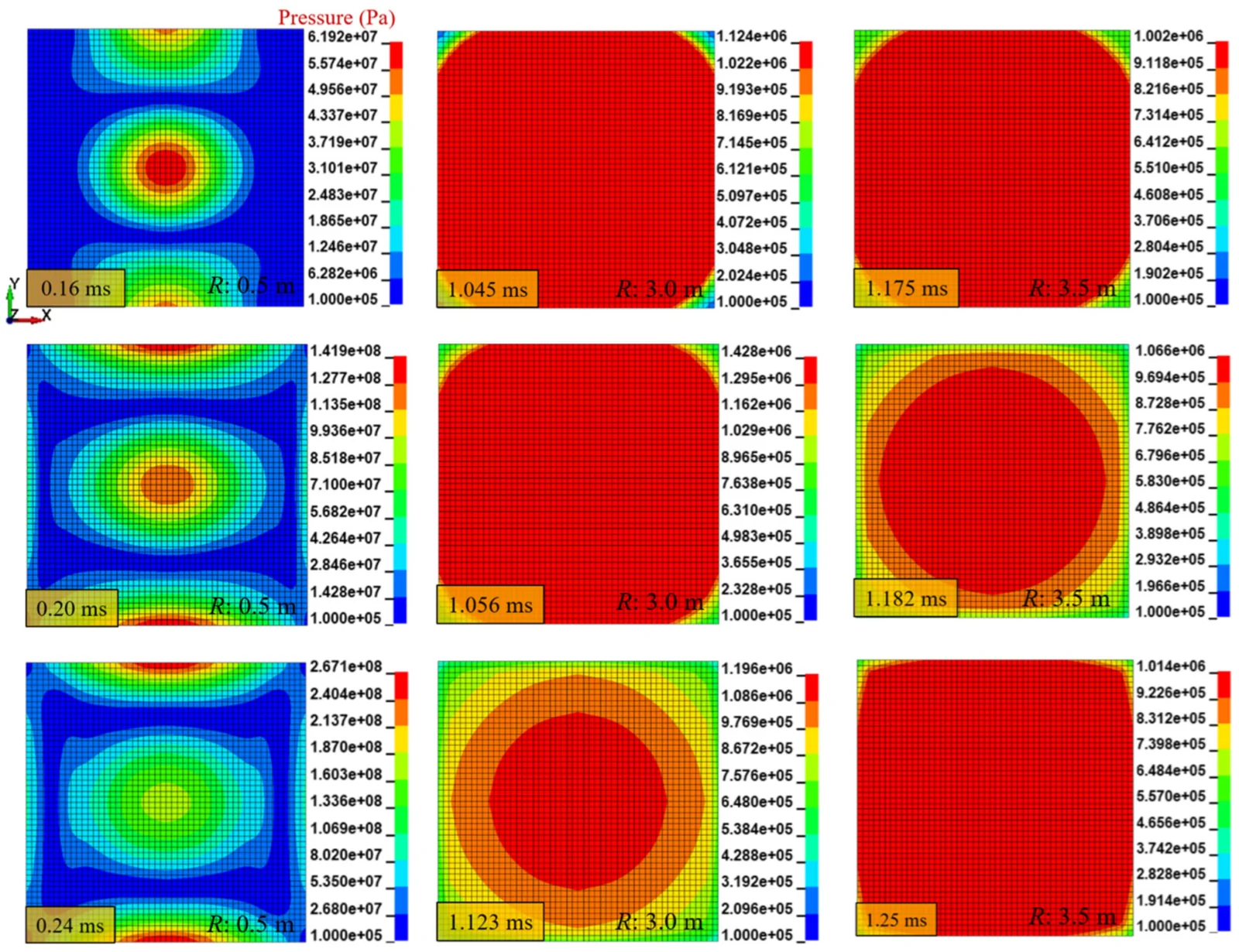
Incident blast pressure contours on laminate surface (plan view) under close-in and near-field blasts.

The incident blast pressure distribution over laminate surface has been investigated in this section through representative spatial contour curves as presented in Figure 11 for the near occurrence of peak pressure for close-in (*R*: 0.5m) and near-field (*R*: 3.0 m, 3.5 m) blasts for 50 kg TNT explosive charge. For *R*: 0.5 m, pressure distribution over the laminate was derived for time instants of 0.16 ms, 0.2 ms, 0.24 ms near to peak pressure occurrence time at 0.17 ms; for *R*: 3.0 m, pressure contours are derived for 1.045 ms, 1.056 ms, and 1.123 ms near to peak pressure occurrence time at 1.07 ms; and for *R*: 3.5 m, pressure contour curves are derived for 1.175 ms, 1.182 ms, and 1.25 ms near peak pressure occurrence at 1.21 ms at the laminate centre. Under close-in blast, contour curves indicate that the laminate centre is subjected to maximum pressure concentration initially, and after attainment of peak pressure at the centre, the pressure concentration is observed at both the centre and edge locations across the y direction. At 0.24 ms, the maximum pressure is observed only at the edges across the y direction. For near-field blast case I, of *R*: 3.0 m, unlike close-in blast, the maximum pressure is seen to be evenly distributed almost across entire laminate surface at 1.045 ms and 1.056 ms, while at 1.123 ms, the maximum pressure starts concentrating towards the centre radially. For *R*: 3.5 m, almost the entire laminate surface is subjected to maximum pressure at 1.175 ms. Afterwards, the maximum pressure starts concentrating towards the centre radially as observed at 1.182 ms; thereafter, the pressure again distributes across surface, as evident from the pressure contour curves derived at 1.25 ms.

For all blast scenarios, the lowest pressure is observed to be 100 kPa, which is atmospheric pressure. Since the pressures observed for three time instants are near to the peak blast pressure, the laminate centre is subjected to respective blast scenarios, and the pressures remain above the atmospheric pressure level, which is evident from the contours, as the curves do not indicate any suction or negative pressure in any of the blast cases.

#### 4.6.2. Laminate Deformation near Peak Deflection Occurrence Time

The state of laminate deformation was analysed through representative spatial contour curves for stiffened laminates under the close-in blast condition. Two separate parametric analyses were performed by (i) keeping the stiffener depth ($d_{s}$) constant while varying the stiffener thickness ($t_{s}$) and (ii) keeping the stiffener thickness constant while varying the stiffener depth. In both the parametric cases, deflection contours are presented for stiffened-${\left[0/90\right]}_{2}$ with stiffener configuration S2.

[a] Parametric case-(i)

**Figure 12:**
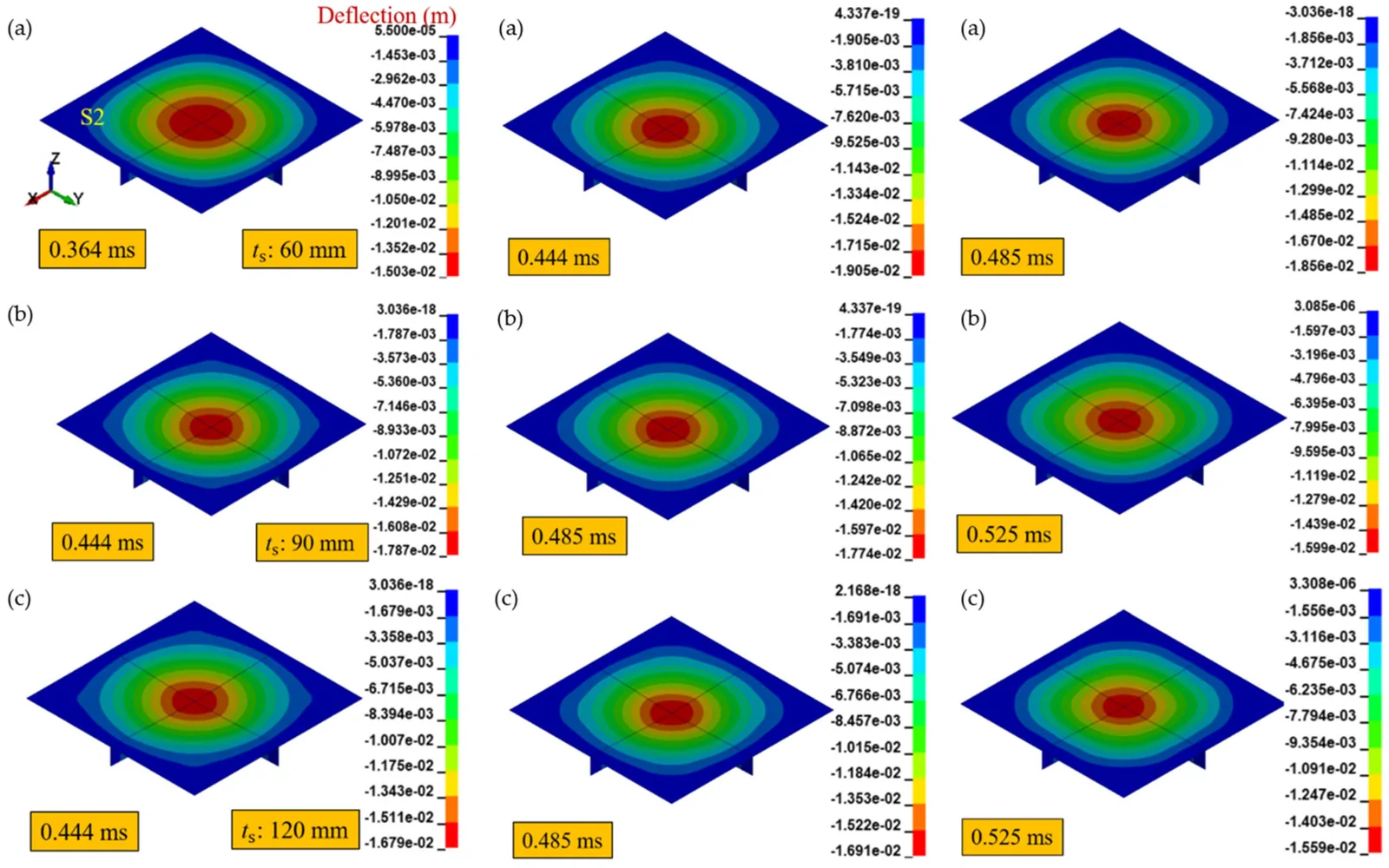
Deflection contour of laminate-${\left[0/90\right]}_{2}$ at $t_{s}$: (**a**) 60 mm, (**b**) 90 mm, and (**c**) 120 mm for uniform $d_{s}$: 100 mm under close-in blast condition.

Representative contours are derived for three $t_{s}$ values of 60 mm, 90 mm, and 120 mm while maintaining $d_{s}$ at 100 mm for laminate ${\left[0/90\right]}_{2}$ bi-directionally stiffened along the x and y directions (S2), as presented in Figure 12. For $t_{s}$: 60 mm, deflection contours are determined for time instants of 0.364 ms, 0.444 ms, and 0.485 ms, near to peak central deflection occurrence at 0.4 ms; for $t_{s}$: 90 mm, 120 mm, the deflection contour curves are analysed at 0.444 ms, 0.485 ms, and 0.525 ms, near to peak central deflection occurrence at approximately 0.46 ms. For all the three $t_{s}$ configurations, the contour curves at three time instants indicate that the laminate with S2 stiffening undergoes maximum deformation at the centre and the centre-adjacent areas of the laminate surface in the radial directions. The analysis further indicates that maximum deflection consistently decreases with increasing $t_{s}$.

[b] Parametric case-(ii)

**Figure 13:**
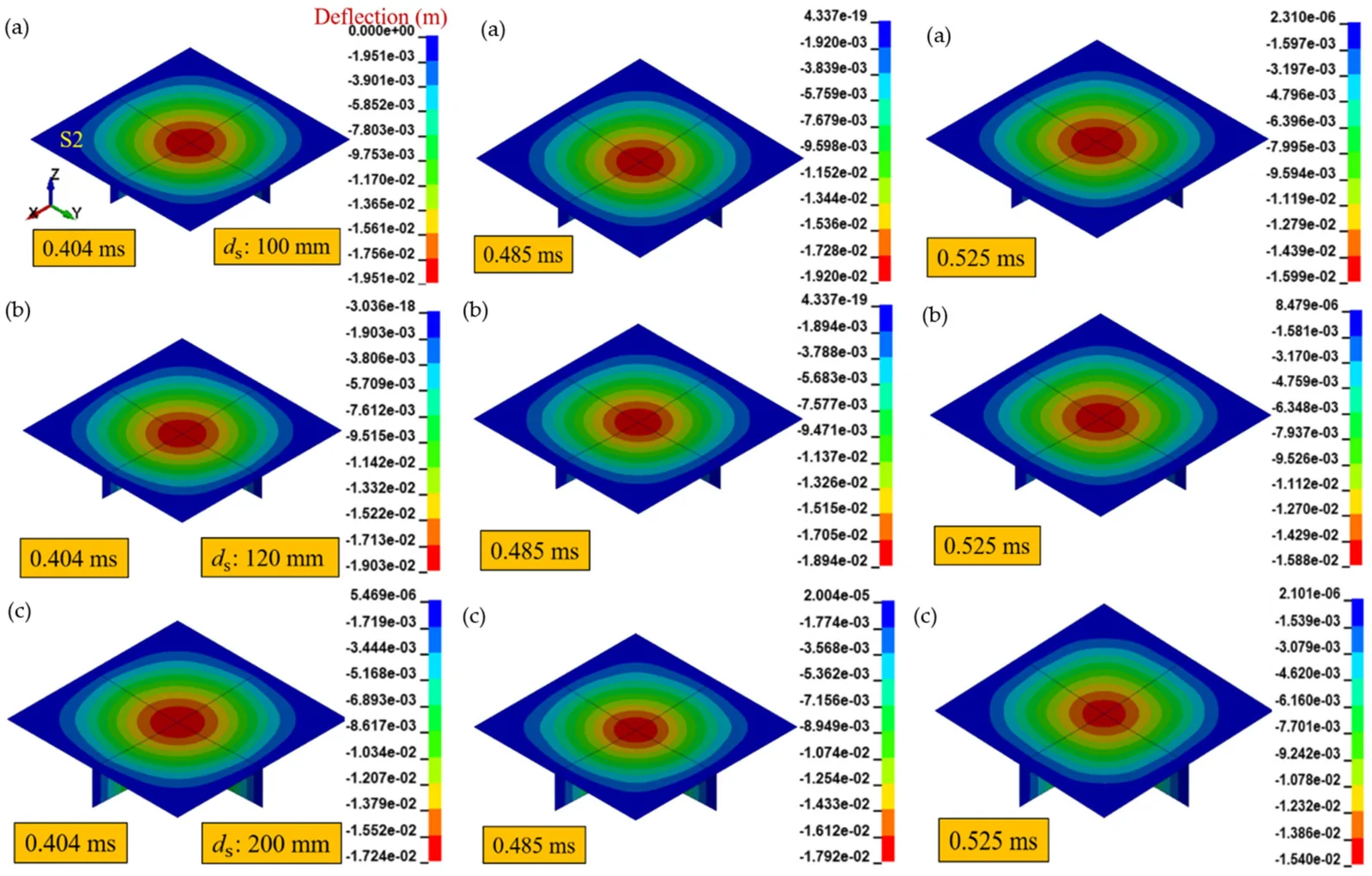
Deflection contour of laminate-${\left[0/90\right]}_{2}$ at $d_{s}$: (**a**) 100 mm, (**b**) 120 mm, and (**c**) 200 mm for uniform $t_{s}$: 30 mm under close-in blast condition.

Representative contour curves are analysed for three $d_{s}$ values of 100 mm, 120 mm, and 200 mm while maintaining $t_{s}$ at 30 mm for laminate ${\left[0/90\right]}_{2}$ bi-directionally stiffened along the x and y directions (S2), as presented in Figure 13. For $d_{s}$: 100 mm, 120 mm, and 200 mm, the deflection contour curves are evaluated at 0.404 ms, 0.485 ms, and 0.525 ms, respectively, near to the occurrences of peak deflection for the respective $d_{s}$ configuration. Here, the contour curves indicate that the centre and the centre-adjacent areas of the laminate undergo maximum deflections at the observed time instants with the S2 stiffener design for the three $d_{s}$ configuration cases. The curves further indicate decreasing maximum laminate deflection with increasing $d_{s}$.

Overall, the results reveal that variation in $t_{s}$ reduces the maximum deflection effectively as compared to the variation in $d_{s}$. In particular, considering two parametric scenarios of stiffener cross-section of (a) $t_{s}$ 120 mm, $d_{s}$ 100 mm and (b) $t_{s}$ 30 mm, $d_{s}$ 120 mm, the maximum deflections are considerably fewer for scenario (a) as compared to scenario (b) at common time instants of 0.485 ms and 0.525 ms, indicative that, in terms of reduction in maximum deflection under close-in blast load, as a critical stiffener parameter, $t_{s}$ has a more pronounced effect as compared to $d_{s}$ for S2-stiffened laminates.

## 5. Conclusions

Polymer-based composite laminates are increasingly employed in aeronautical structures owing to their high stiffness-to-weight ratio, favourable elastic characteristics, and energy dissipation capability. This study investigated the nonlinear dynamic response of laminated FRP plates under close-in and near-field spherical blast loading using a validated Coupled Eulerian–Lagrangian (CEL) finite element framework. For blast simulations especially for close-in blast, the CEL algorithm gives better prediction as compared to CONWEP formulation which is based on Kingery-Bulmash theory (Kingery and Bulmash 1984 [55]). The present analysis was primarily aimed at investigating the elastic structural response of the FRP plates under blast loading rather than to quantitively predict strength-based composite FRP laminate failure. The laminates were modelled using an equivalent single-layer approach with orthotropic properties from the literature, selected based on the reported spatial and temporal convergence studies, and the numerical models were extensively validated against benchmark experimental and analytical results to ensure reliability of the predictive numerical framework under a high-strain-rate loading (Johnson 1983 [56]) scenario. The study focused on the evaluation of instantaneous response of light-weight, high strength-to-weight-ratio FRP plates, which could be used as an enclosure (box) wall in the next stage of advanced research, under close-in blast loading, and systematically examined laminates’ response to different laminate stacking sequences and bottom-mounted stiffener configurations by varying the stiffener depth and thickness to propose an effective blast-resistant design. Based on the numerical findings, the following key conclusions are reported.

1. Under unstiffened conditions, the four-layer unsymmetric angle-ply laminate exhibited the highest blast resistance under close-in loading, with the lowest peak deflection of 22.28 mm, representing reductions of 3.0–6.55% relative to the other laminate configurations. In contrast, under near-field blast loading, the four-layer unsymmetric cross-ply laminate consistently produced the lowest peak deflections (1.23, 0.522, and 0.214 mm at stand-off distances of 2.4, 3.0, and 3.5 m, respectively), with reductions of up to 7.94% compared with the remaining laminates.
2. Although the four-layer unstiffened unsymmetric angle-ply laminate exhibited the lowest peak deflection under close-in blast loading, it is observed that with increasing ply count, laminate becomes more blast-resistant with a cross-ply configuration. From five to eight plies, the cross-ply laminate shows peak deflections of 21.42, 21.00, 20.62, and 20.24 mm, corresponding to 2.10–4.59% lower values than its angle-ply counterparts. It is further observed that eight-layered cross-ply achieves up to an 11.81% reduction in peak deflection relative to the four-ply configuration.
3. Among the four stiffener layouts, the S2 configuration provided the highest blast resistance, particularly when combined with the four-layer unsymmetric cross-ply laminate under close-in loading. Relative to the unstiffened laminate (22.95 mm peak deflection), the S2 configuration reduced the peak deflection by up to 25.80% with increasing stiffener thickness (30–120 mm) at a constant depth of 100 mm and by up to 21.57% with increasing stiffener depth (70–200 mm) at a constant thickness of 30 mm.
4. Peak deflections decreased progressively with increasing stiffener depth and thickness for all laminate and stiffener configurations. The maximum reduction was achieved with a stiffener depth of 200 mm and a thickness of 120 mm. Under the present scope of research, given the nature of numerically derived close-in blast, lamina stacking sequences, and stiffener configurations, stiffener thickness is observed to be a more critical parameter than stiffener depth in influencing blast-loaded peak responses of stiffened FRPs

## Figures and Tables

**Table 1:** Full scale and quarter-symmetric FE model of laminate and applied symmetric boundary.

Stiffener configuration			
S1	S2	S3	S4
Isometric view of stiffened laminate model			
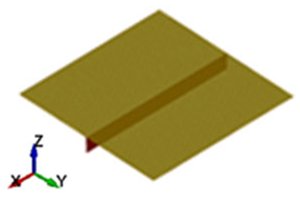	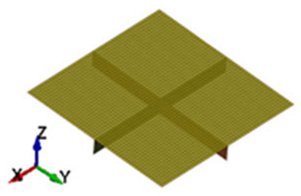	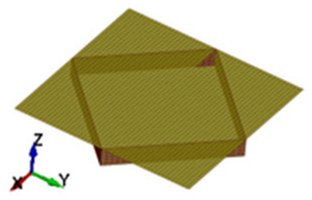	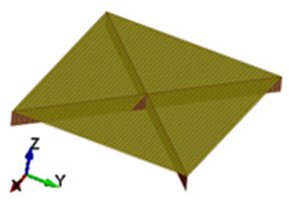
Quarter-symmetric stiffened laminate model with symmetric edges			
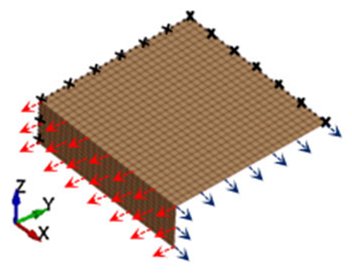	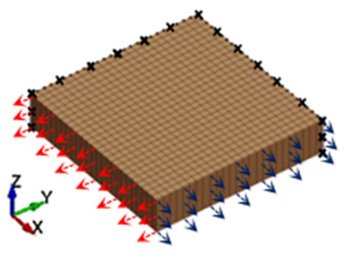	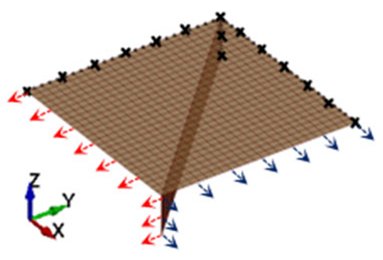	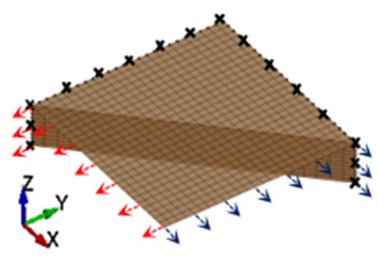
Boundary conditions in quarter-symmetric model			
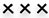 ➢ Fixed	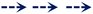 ➢ x–symmetry		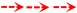 ➢ y–symmetry

**Table 2:** Material and state parameters for explosives (Lee et al. 1973 [44]; Dobratz 1981 [45]; Giam et al. 2020 [46]).

MAT_HIGH_EXPLOSIVE_BURN (kg, m, s, N, Pa)						
Density ρ (kg/m^3^)		Detonation velocity D (m/s)			Chapman-Jouguet pressure $P_{\mathrm{CJ}}$ (GPa)	
1630		6930			21	
EOS_JWL						
A (GPa)	B (GPa)	$R_{1}$	$R_{2}$	ω	E (GPa)	V
371.2	3.231	4.15	0.95	0.3	7	1

**Table 3:** Material and state parameters for air (Bae and Zakki 2011 [50]; Marrs and Heiges 2014 [51]; Dolce et al. 2010 [52]).

$\rho_{0}$	Pressure cutoff	Viscosity	$C_{0}$	$C_{1}$	$C_{2}$	$C_{3}$	$C_{4}$	$C_{5}$	$C_{6}$	$E_{0}$	$V_{0}$
(kg/m^3^)	(Pa)	(N-s·m^−2^)	(Pa)	(Pa)	(Pa)	(Pa)				(Pa)	
1.29	$-1\times 10^{-10}$	$2\times 10^{-5}$	0	0	0	0	0.4	0.4	0	$2\times 10^{5}$	1

**Table 4:** Composite material properties (Kazancı and Mecitoğlu, Z. 2008 [53]; Upadhyay et al. 2011 [54]).

ρ (kg/m^3^)	$E_{1}$ (Pa)	$E_{2}$ (Pa)	$\nu_{21}$	$\nu_{31}$	$\nu_{32}$
1443	1.324 × 10^11^	1.08 × 10^10^	0.0196	-	-
$G_{12}$ (Pa)	$G_{23}$ (Pa)	$G_{13}$ (Pa)	AOPT		
5.6 × 10^9^	11.2 × 10^9^	5.6 × 10^9^	(user-defined)		

**Table 5:** Peak normal stresses at the top and bottom faces of the symmetric cross and angle ply.

Blast standoff distance (R)											
R: 0.5 m			R: 2.4 m			R: 3.0 m			R: 3.5 m		
(Pa) (×10^9^)			Peak stress (Pa) (×10^8^)			Peak stress (Pa) (×10^7^)			Peak stress (Pa) (×10^7^)		
Laminate code	Surface		Laminate code	Surface		Laminate code	Surface		Laminate code	Surface	
	Top	Bottom		Top	Bottom		Top	Bottom		Top	Bottom
${\left[\pm 45\right]}_{s}$	−1.34	1.44	${\left[\pm 45\right]}_{s}$	−0.79	0.80	${\left[\pm 45\right]}_{s}$	−3.41	3.46	${\left[\pm 45\right]}_{s}$	−1.45	1.45
${\left[0/90\right]}_{s}$	−1.21	1.50	${\left[0/90\right]}_{s}$	−0.76	0.77	${\left[0/90\right]}_{s}$	−3.28	3.29	${\left[0/90\right]}_{s}$	−1.40	1.40

**Table 6:** Peak deflection and its occurrence time of unstiffened FRP laminates.

	Laminate	${\left[\pm 45\right]}_{s}$		${\left[0/90\right]}_{s}$		${\left[\pm 45\right]}_{2}$		${\left[0/90\right]}_{2}$	
Standoff (*R*)		Deflection (mm)	Time (ms)	Deflection (mm)	Time (ms)	Deflection (mm)	Time (ms)	Deflection (mm)	Time (ms)
0.5 m		23.20	0.44	23.74	0.46	22.28	0.44	22.95	0.43
3.0 m		0.56	1.75	0.54	1.76	0.53	1.75	0.52	1.74
3.5 m		0.23	2.27	0.22	2.27	0.22	2.26	0.21	2.24

**Table 7:** Close-in blast-induced peak deflections of unstiffened laminates for different ply counts.

Laminate (170 mm Thick)	Peak Deflection (m)			
	5 Layers	6 Layers	7 Layers	8 Layers
0/90	0.02142	0.0210	0.02062	0.02024
±45	0.02206	0.02144	0.02133	0.02117

**Table 8:** Lamina sequences of cross and angle ply for four different ply counts.

Laminate Code	Lamina Sequence	Lamina Count
${\left[\left(0/90\right)/\bar{0}\right]}_{s}$	$0^{{}^{\circ}}/90^{{}^{\circ}}/0^{{}^{\circ}}/90^{{}^{\circ}}/0^{{}^{\circ}}$	Five
${\left[\pm 45/\bar{45}\right]}_{s}$	$45^{{}^{\circ}}/{-45}^{{}^{\circ}}/45^{{}^{\circ}}/{-45}^{{}^{\circ}}/45^{{}^{\circ}}$	
${\left[0/90\right]}_{3}$	$0^{{}^{\circ}}/90^{{}^{\circ}}/0^{{}^{\circ}}/90^{{}^{\circ}}/0^{{}^{\circ}}/90^{{}^{\circ}}$	Six
${\left[\pm 45\right]}_{3}$	$45^{{}^{\circ}}/{-45}^{{}^{\circ}}/45^{{}^{\circ}}/{-45}^{{}^{\circ}}/45^{{}^{\circ}}/{-45}^{{}^{\circ}}$	
${\left[\left(0/90/0\right)/\bar{90}\right]}_{s}$	$0^{{}^{\circ}}/90^{{}^{\circ}}/0^{{}^{\circ}}/90^{{}^{\circ}}/0^{{}^{\circ}}/90^{{}^{\circ}}/0^{{}^{\circ}}$	Seven
${\left[\left(\pm 45/45\right)\bar{-45}\right]}_{s}$	$45^{{}^{\circ}}/{-45}^{{}^{\circ}}/45^{{}^{\circ}}/{-45}^{{}^{\circ}}/45^{{}^{\circ}}/{-45}^{{}^{\circ}}/45^{{}^{\circ}}$	
${\left[0/90\right]}_{4}$	$0^{{}^{\circ}}/90^{{}^{\circ}}/0^{{}^{\circ}}/90^{{}^{\circ}}/0^{{}^{\circ}}/90^{{}^{\circ}}/0^{{}^{\circ}}/90^{{}^{\circ}}$	Eight
${\left[\pm 45\right]}_{4}$	$45^{{}^{\circ}}/{-45}^{{}^{\circ}}/45^{{}^{\circ}}/{-45}^{{}^{\circ}}/45^{{}^{\circ}}/{-45}^{{}^{\circ}}/45^{{}^{\circ}}/{-45}^{{}^{\circ}}$	

**Figure A1:**
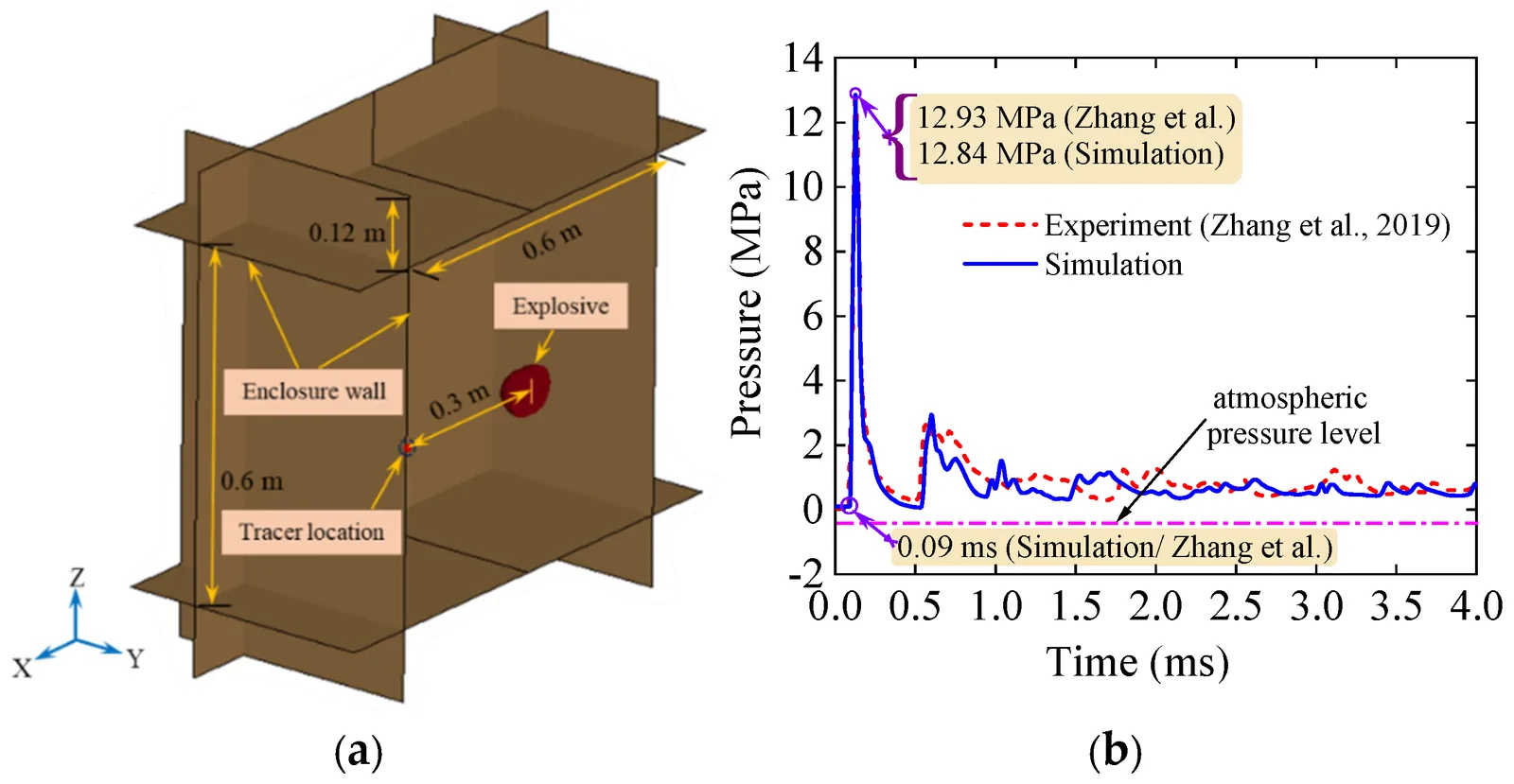
(**a**) Section view of CEL enclosure model and (**b**) validation of predicted confined close-in blast pressure with reported experimental study by Zhang et al. (2019) [58].

**Figure A2:**
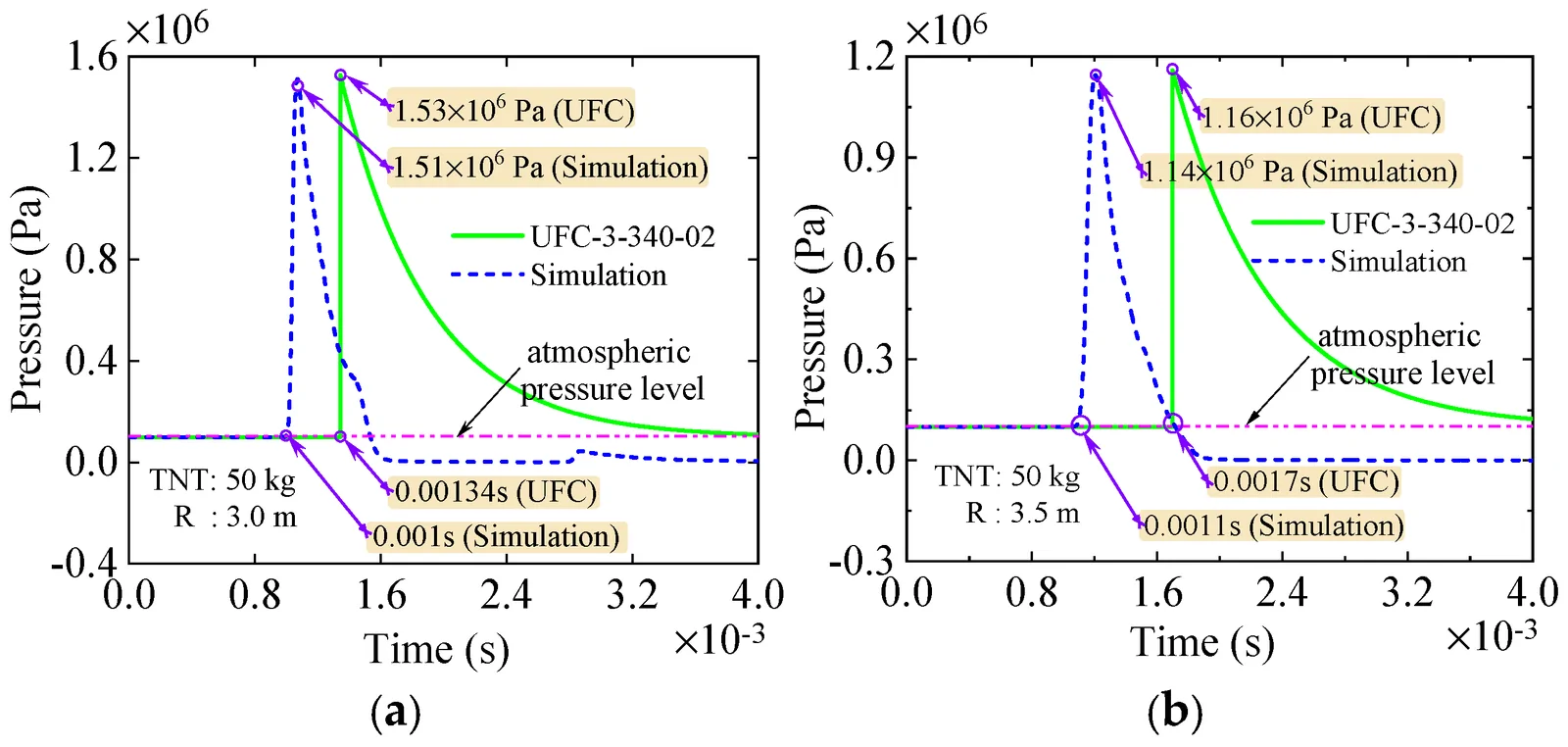
Comparison of predicted near-field blast load with UFC for standoffs of (**a**) 3.0 m and (**b**) 3.5 m.

**Figure A3:**
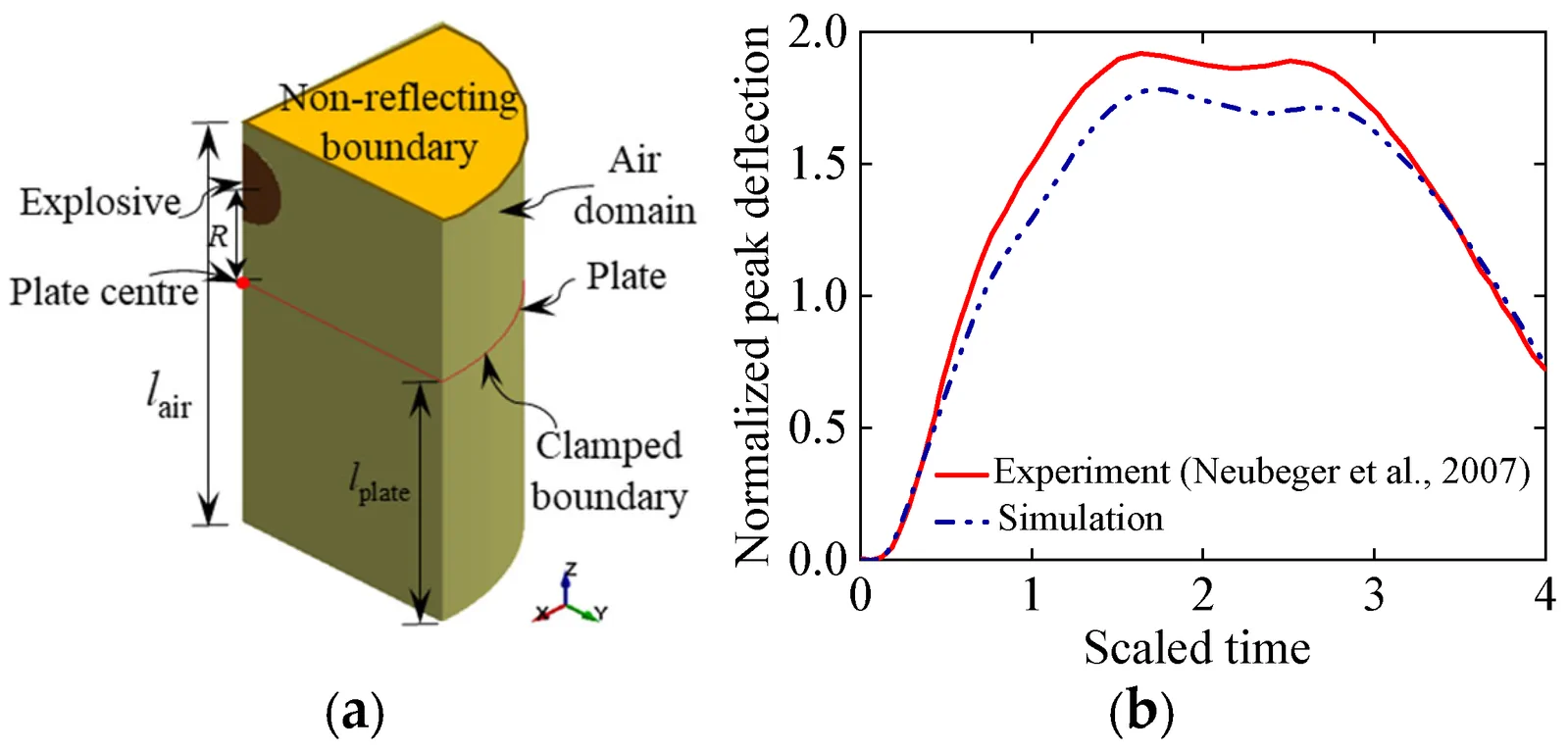
(**a**) Quarter-symmetric CEL model and (**b**) validation of predicted response with reported experimental study by Neuberger et al. (2007) [62] under close-in detonation.

**Figure A4:**
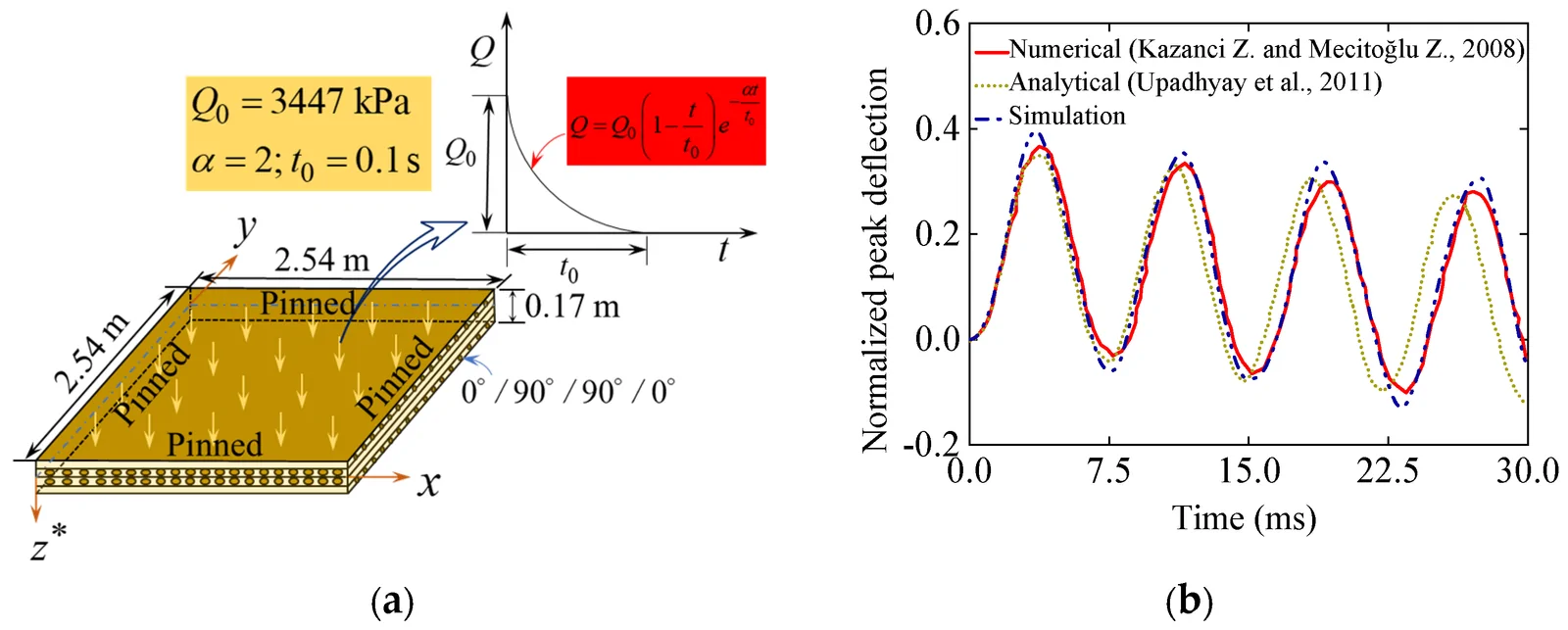
(**a**) FRP plate configuration with applied blast load profile and (**b**) validation of predicted response with reported study (Kazancı and Mecitoğlu 2008 [53]; Upadhyay et al. 2011 [54]).

**Figure A5:**
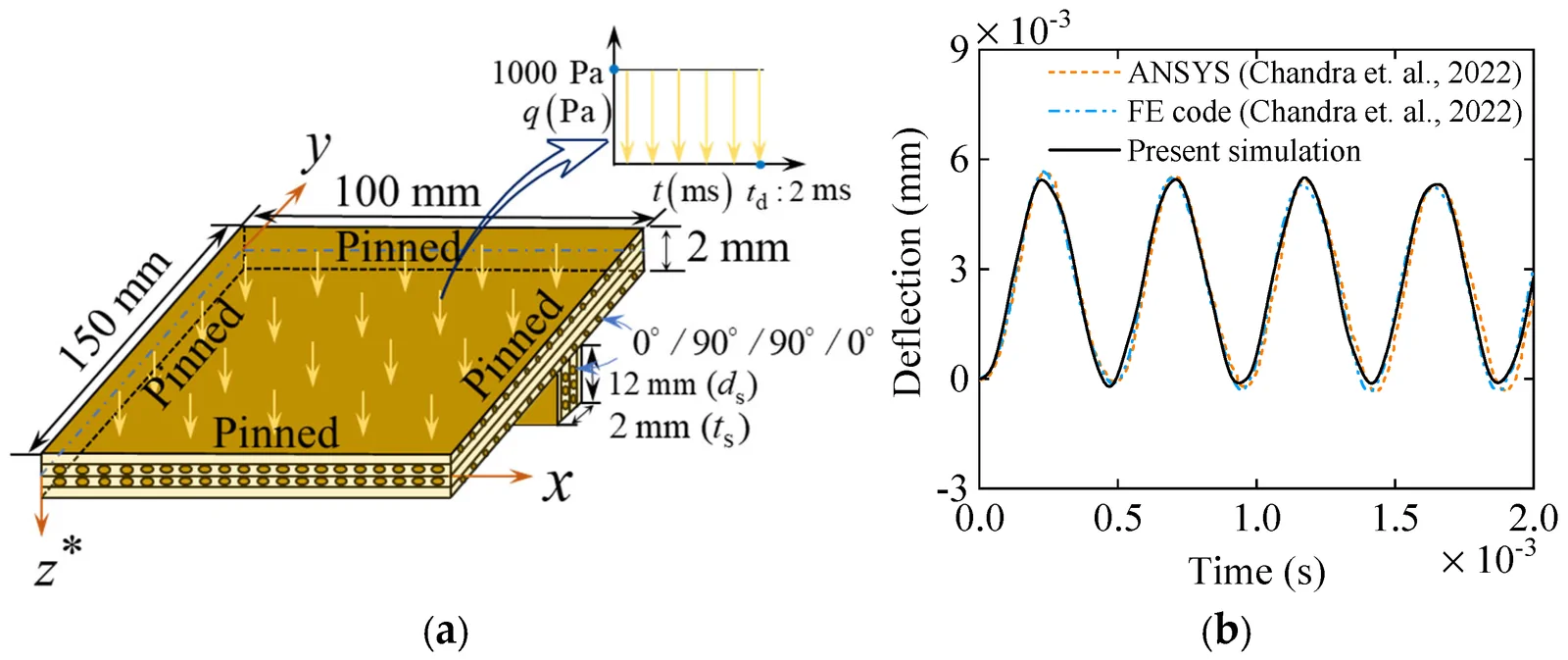
(**a**) FRP laminate configuration with rectangular pulse load and (**b**) validation of predicted response with reported study (Chandra et al. 2022 [65]).

## Data Availability

Some or all data, models, or code that support the findings of this study are available from the corresponding author upon reasonable request.

## Appendix A

### Appendix A.1. Validation of Numerically Predicted Blast Pressure Against Experimental Observation Under Close-In Internal Blast Using CEL Numerical Platform

This appendix reports the validation study for the adopted explosive material model and equation-of-state parameters for simulating close-in blast loading by comparing the predicted blast pressure–time history with published experimental data (Lu et al. 2021 [57]; Zhang et al. 2019 [58]). The validation model reproduces the experimental box-shaped enclosure containing a centrally located 98.44 gm spherical TNT charge, with the pressure history recorded at the centre of the enclosure wall, as illustrated in Figure A1a. The explosive was modelled using MAT_HIGH_EXPLOSIVE_BURN and EOS_JWL, while the transient pressure history was obtained using the DATABASE_TRACER option with a fixed tracer to minimise the influence of reflected waves. Figure A1b compares the predicted and experimental pressure–time histories. The numerical model accurately predicts the blast-wave arrival time (0.09 ms) and the peak pressure with only 0.69% deviation from the experimental result while closely reproducing the pressure decay trend. These results confirm the accuracy of the adopted CEL framework and explosive material model for simulating close-in blast loading.

### Appendix A.2. Validation of Numerically Predicted Blast Pressure Against Benchmark Standard Under Near-Field Free Air Blast Using CEL Numerical Platform

This appendix validates the adopted explosive material model and state parameters for near-field blast simulation by comparing the predicted blast pressure–time histories with the benchmark standards in UFC 3-340-02 (Acosta 2011 [59]; Karlos and Solomos 2014 [60]). Numerical simulations were performed for 50 kg TNT charges at stand-off distances of 3.0 and 3.5 m using the proposed CEL framework. The corresponding code-based pressure histories were constructed from the UFC peak overpressure, arrival time, positive-phase duration, and decay coefficient associated with each scaled distance (Karlos et al. 2016 [61]). Figure A2 compares the numerical and UFC pressure–time histories, highlighting the arrival time and peak pressure for both blast scenarios. The predicted peak pressures show excellent agreement with the UFC estimates, with deviations of only 1.31% and 1.72% for stand-off distances of 3.0 and 3.5 m, respectively. Although the numerical arrival times differ from the code predictions by 25.37% and 34.71%, respectively, they remain within acceptable engineering limits. Overall, the comparison confirms the accuracy of the adopted explosive model and CEL framework for near-field blast simulation.

### Appendix A.3. Validation of Dynamic Response of CEL-Model Under Close-In Blast Against Experiment

This validation study was performed to assess the reliability of the CEL algorithm in deriving structural response under close-in blast with good accuracy. For evaluation, the CEL algorithm was employed in reproducing structural (panel) elastic response under close-in blast as experimentally investigated by Neuberger et al. (2007) [62]. The validation considered the deflection–time history of a clamped circular rolled homogeneous armour (RHA) steel plate (1 m diameter) subjected to a W 50 kg TNT charge at R = 0.5 m. The numerical model employed MAT_PLASTIC_KINEMATIC with Cowper–Symonds strain-rate effects (Upadhyay et al. 2011 [54]; Giam et al. 2020 [46]; Marrs and Heiges 2014 [51]; Orton et al. 2013 [34]; Nurick et al. 1995 [63]; Kadid 2008 [64]), while the explosive was represented using MAT_HIGH_EXPLOSIVE_BURN and EOS_JWL. A quarter-symmetric CEL model was developed with optimized air-domain dimensions, shell elements for the steel plate, solid elements for the Eulerian air, and fluid–structure interaction established through CONSTRAINED_LAGRANGE_IN_SOLID. The armour steel plate is modelled using a four-noded, square fully integrated shell element with a size of 20 mm in the central zone using a shape mesher circular shell geometry with the LS-DYNA platform, while the air is modelled using an eight-noded solid element with a size of 20 mm in the x–y plane and 20 mm in the z-direction. The overall air-domain depth ($l_{\mathrm{air}}$) is optimized to 2 m, while the plate is positioned at a height ($l_{\mathrm{plate}}$) of 1.2 m above the air bottom. As shown in Figure A3, the predicted deflection–time history agrees well with the experimental measurements, confirming the accuracy and robustness of the proposed CEL framework for simulating close-in blast-induced structural response.

### Appendix A.4. Validation of Dynamic Response of Unstiffened FRP Laminated Composite Plate Under Exponentially Decaying Pulse Loading

This validation is particularly aimed at assessing the reliability of the adopted FRP laminate modelling framework in simulating elastic deformation of FRP laminate under blast with good accuracy. To evaluate the fidelity of the FRP modelling process, this section validates the laminated-composite-plate-model response against the analytical and numerical results reported by Kazancı and Mecitoğlu (2008) [53] and Upadhyay et al. (2011) [54] under an exponentially decaying blast load. The material properties reported by Upadhyay et al. (2011) [54] are adopted in the present model. As shown in Figure A4, a four-layer symmetric cross-ply laminate (2.54 × 2.54 × 0.17 m) is modelled using an equivalent single-layer shell formulation with PART_COMPOSITE_LONG, where the lamina properties are assigned sequentially through the thickness. The laminate is discretized using four-noded fully integrated shell elements (30 mm) and subjected to simply supported boundary conditions. An exponentially decaying pulse pressure load with a peak overpressure of $Q_{0}$ = 3447 kPa (an exponentially decaying pulse function of decay constant α over a period $t_{0}$ as per Figure A4a) is applied to the top surface using LOAD_CURVE and LOAD_BLAST_SEGMENT_SET (Ls-PrePost version 4.8). The normalized central deflection predicted by the present model shows good agreement with the reported analytical and numerical results (Figure A4b), confirming the accuracy of the adopted laminate modelling approach.

### Appendix A.5. Validation of Dynamic Response of Stiffened FRP Laminated Composite Plate Under Constant Pulse Load

This validation studies the fidelity of the modelling framework in determining stiffened-FRP laminate response and assessing stiffener-plate connection performance under sudden applied (impact) constant (rectangular) pulse load with good accuracy.

The present section compares the elastic response of a unidirectionally stiffened laminated-composite-plate model with the available study by Chandra et al. (2022) [65]. As shown in Figure A5a, both the plate and centrally attached bottom stiffener comprise four-layer symmetric cross-ply laminates modelled using the equivalent single-layer approach with PART_COMPOSITE_LONG. Simply supported boundary conditions are applied along all plate edges. A uniform pulse load of constant magnitude (q) (1000 Pa) is applied to the laminate top surface using LOAD_CURVE and LOAD_BLAST_SEGMENT_SET. The plate (20 × 20) and stiffener (20 × 4) are discretized using four-noded fully integrated shell elements. The predicted central deflection agrees closely with the published results (Figure A5b), confirming the accuracy of the adopted modelling approach for stiffened laminated composite plates.
